# Leukemia inhibitory factor receptor homodimerization mediated by acetylation of extracellular lysine promotes prostate cancer progression through the PDPK1/AKT/GCN5 axis

**DOI:** 10.1002/ctm2.676

**Published:** 2022-02-16

**Authors:** Yufeng Ding, Jialiang Shao, Tiezhu Shi, Hua Yu, Xiang Wang, Honggang Chi, Xiongjun Wang

**Affiliations:** ^1^ School of Life Sciences Guangzhou University Guangzhou China; ^2^ Department of Urology Shanghai General Hospital Shanghai Jiaotong University Shanghai China; ^3^ Department of Traditional Chinese Medicine The First Dongguan Affiliated Hospital of Guangdong Medical University Dongguan China

## Abstract

**Background:**

Prostate cancer (PCa), an inert tumour, has a long progression period, but valid biomarkers and methods for effectively and sensitively monitoring PCa progression are lacking, prompting us to identify new predictors for diagnosis and prognosis. Posttranslational modifications characterizing receptor activation are considered potentially strong indicators of disease progression.

**Methods:**

The posttranscriptional regulation of leukaemia inhibitory factor receptor (LIFR) and its novel downstream signalling activity in PCa were studied using liquid mass spectrometry, genetically engineered mouse (GEM) models, organoid assays, lentivirus packaging, infection and stable cell line construction.

**Results:**

In this study, the level of acetylated K620 on LIFR in its extracellular domain was shown to predict the progression and prognosis of PCa. In PCa cells, LIFR‐K620 acetylation is reversibly mediated by GCN5 and SIRT2. GEM experiments and organoid assays confirmed that the loss of LIFR‐K620 acetylation inhibits PCa progression. Mechanistically, K620 acetylation facilitates LIFR homodimerization and subsequently promotes LIFR‐S1044 phosphorylation and activation, which further recruits PDPK1 to activate AKT signalling and sequentially enhances the GCN5 protein level to sustain the protumour level of LIFR‐K620 acetylation by preventing GCN5 degradation via CRL4^Cdt2^ E3 ligase.

**Conclusions:**

Acetylation of extracellular K620 on LIFR reinforces its homodimerization and integrates the activities of PDPK1, AKT, GSK3β and GCN5 to form a novel positive feedback loop in PCa; this modification is thus a promising biomarker for monitoring PCa progression.

## Graphical abstract

We first demonstrated that LIFR homodimer formation mediated by K620 acetylation is upregulated in the prostate epithelium in an AKT‐dependent manner and is indispensably required for the malignancy of PTEN‐null tumors. Based on this study, the acetylation of LIFR‐K620 can be used as a biomarker for prognosis in prostate cancer patients for non‐invasive testing, and also as a potential therapeutic target.

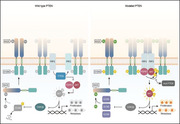

## 1 BACKGROUND

Prostate cancer (PCa) initiates slowly from a prostatic intraepithelial neoplasm (PIN) to adenocarcinoma and subsequently metastatic cancer. Many genetic and epigenetic mutations have been shown to promote PCa progression, such as TP53 mutation and Myc amplification.^1^ However, these alterations are not conveniently targeted or detected; hence, there is an impending clinical need for more effective and convenient PCa biomarkers that facilitate the monitoring and treatment of PCa progression. Specifically, cancer biomarkers that can be measured noninvasively (blood or urine) is of great significance since these types of samples can be noninvasively acquired from patients during the whole course of the disease and would reflect the progression of tumours with more convenience and accuracy.

Membrane receptors and their posttranslational modifications often herald the transduction of ligand signals and, when dysfunctional, promote disease progression, which may provide more effective targets for therapy.^2^, ^3^, ^4^, ^5^ Some receptors are soluble in blood, such as the soluble IL6 receptor and urokinase plasminogen activator receptor, leading these proteins to serve reliable biomarkers that can be easily detected.^6^, ^7^, ^8^, ^9^, ^10^ Therefore, based on our previous study, we focused on leukaemia inhibitory factor receptor (LIFR) translational modification to determine whether LIFR‐S1044 phosphorylation augmented the oncogenic signal to alpha serine/threonine‐protein kinase (AKT) and promoted PCa progression.^11^

However, S1044 is located in the intracellular domain of LIFR and cannot be modified for the clinical liquid biopsy of PCa. Acetylation on lysine (K) residues of soluble proteins is stable and has been proven to be detectable.^11^ Hence, we investigated acetylation modifications on the extracellular domain of LIFR and observed that LIFR‐K620 was acetylated by histone acetyltransferase KAT2A (GCN5), is soluble in the blood and was greatly correlated with clinical PCa progression.

Based on these data, LIFR‐K620 acetylation is a good biomarker for the diagnosis of PCa. Next, we observed that K620 acetylation promoted LIFR homodimerization and increased S1044 phosphorylation, thus enhancing AKT activation and promoting PCa progression. Mechanistically, AKT maintains the protein stability of GCN5 by antagonizing glycogen synthase kinase‐3β (GSK3β)‐mediated CRL4^Cdt2^ E3 ligase‐dependent degradation of GCN5. The increased levels of GCN5 lead to direct increases in acetylated LIFR‐K620, which further promotes AKT activation and PCa progression in a positive feedback loop. Our study identifies the extracellular lysine acetylation of LIFR as an additional regulator of AKT activation and an effective biomarker in the blood of patients with PCa.

1 **HIGHLIGHT**

- LIFR‐K620ac mediates the homodimmer formation of LIFR that promotes LIFR‐S1044 phosphorylation and further recruits PDPK1 to activate AKT signalling.
- LIFR‐K620ac is acetylated by GCN5 and de‐acetylated by SIRT2.
- In turn, activation of AKT signalling enhances GCN5 protein levels to feedback sustain the tumourigenic level of LIFR‐K620 acetylation by preventing GCN5 degradation.

## 2 METHODS

### 2.1 Human tissue microarray analysis and ethics

The Asian radical prostatectomy cohort and corresponding patient inclusion and exclusion criteria were described previously.^11^ All related information is listed in Table S2. The enrolled patients signed informed consent forms, and this study was approved by the ethics committee at our institution (approval number SHF2019021). Immunohistochemistry (IHC) analysis was performed using anti‐LIFR‐K378ac, anti‐LIFR‐K442ac and anti‐LIFR‐K620ac antibodies (ABclonal Technology, Wuhan, China). The staining intensity was quantified and scored by pathologists who were blinded to the case outcomes. Briefly, quantification of the staining intensity was made based on multireplicative scores of the average staining intensity (0– 3) and the extent of staining (0–3), yielding a 10‐point staining score ranging from 0 (no staining) to 9 (strong staining). A staining score ≤ 4 was defined as low, and a score ≥ 4 was defined as high.

### 2.2 Animal experiments and cell line authentication

All mice were maintained in a specific pathogen‐free facility, and all cell lines were authenticated by American Type Culture Collection or Chinese Academy of Sciences. For the subcutaneous xenograft model, 5 × 10^5^ PC3 cells mixed with Matrigel were injected into the ventral prostates (VPs) of male nude mice. The tumour was measured every 7 days, with tumour volume calculated using the formula 0.52 × *L* (length) × *W* (width)^2^.

For intracardiac injection assays, 1 × 10^6^ PC3 cells were intracardially injected into 6‐ to 8‐week‐old nude mice. A NightOWL II LB 983 Imaging System (Berthold Technologies) was utilized to perform bioluminescent imaging (BLI) experiments, and an X‐ray Radiography System (Faxitron) was used to detect the extent of bone damage. The data were quantified with ImageJ software.

LIFR‐KR mutant mice were constructed by the method described previously.^12^, ^13^ *Pten* flox mice were a gift from Dr. Hong Wu at UCLA.^14^ PB^Cre/+^ (Probasin promoter‐driven Cre recombinase)^15^ and TMPRSS2^Cre‐ERT2‐IRES‐GFP^ (ERT2‐induced TMPRSS2 promoter‐driven Cre recombinase) mice^16^ were established on a C57BL/6 background. All the GEM models used here were backcrossed into a C57BL/6 background for more than five generations to avoid unnecessary variation generated by a mixed background.

### 2.3 Organoid culture and experiments

The lobes of the prostate were isolated from TMPRSS2^Cre‐ERT2‐IRES‐GFP^; *Pten* ^fl/fl^ mice, minced into small pieces (∼1 mm^3^), digested at 37°C on a shaking platform and sorted by GFP expression to isolate prostate luminal cells. Cells were resuspended in phosphate buffered saline (PBS) mixed with Matrigel (BD Biosciences, volume ratio = 1:1), and the suspensions were plated into 24‐well plates covered with prostate organoid culture. Afimoxifene (4‐OHT, Sigma–Aldrich, H7904) was added to the culture to induce CreERT2 and Pten deletion. Then, the packaged lentivirus was added to the organoids, and infected cells were selected by puromycin. The numbers, size and formation efficiency of the organoids were determined on day 10. Urogenital sinus mesenchyme (UGSM) cell isolation and the organoid transplantation assay were performed as previously described.^17^ A total of 1×10^6^ UGSM cells were mixed with 1×10^6^ organoid cells stably expressing luciferase and then used to generate xenografts. The cells were resuspended in Matrigel (BD Biosciences, 1:1) and then injected into the VPs of 6‐week‐old male NSG mice. Two months later, a NightOWL II LB 983 Imaging System (Berthold Technologies) was used to conduct BLI and determine the tumour volumes in mice.

Biopsy specimens from PCa patients were minced into small pieces (∼1 mm^3^) and digested at 37°C on a shaking platform in 5 ml of 5 mg/ml type II collagenase (Invitrogen) in Advanced DMEM/F12 (ADMEM/F12) for 2 h. Dissociated cells were washed and resuspended in PBS mixed with Matrigel (BD Biosciences, volume ratio = 1:1), and the suspensions were plated into 24‐well plates covered with prostate organoid culture. Human PCa organoids were weekly passaged at a 1:3 passage ratio, digested by TrypLE (Sigma–Aldrich) at 37°C for 5 min. The organoids were cultured and collected for Western blotting (WB). The reagents used for the organoid culture are listed in the Supporting information.

### 2.4 IHC and histology

First, 4% paraformaldehyde (PFA) was added to the ventral, dorsolateral, and anterior lobes of prostate to fix the tissues overnight, and then the tissues were paraffin embedded using standard procedures. Then, the tissues were sliced into sections of 7 μm thickness, deparaffinized with dimethyl benzene and dehydrated in a gradient series of ethanol. The sections were heated for 15 min in sodium citrate buffer (Vector Laboratories) for antigen retrieval and then permeabilized in PBS containing 0.1% Triton X‐100 for 10 min before they were blocked with PBS containing 5% goat serum for 1 h. Next, primary antibodies were incubated with the sections at 4°C overnight followed by incubation with secondary antibodies at room temperature for 1 h at an appropriate dilution ratio according to the manufacturer's instructions. The scores and quantification of protein expression were performed by pathologists who were blinded to the case outcomes. The quantification was made based on multi‐replicative scores of the average staining intensity (0– 3) and the extent of staining (0–3), ranging from 0 (no staining) to 9 (strong staining). All analyses were independently conducted by three qualified pathologists. After H&E staining, prostate hyperplasia was determined based on the proliferation of luminal cells with no cytological atypia containing small foci and 2 or 3 layers of cells. Histology was graded as described in a previous report.^18^

### 2.5 Mass spectrometric analysis of LIFR‐associated proteins

Immunoprecipitated DYKDDDDK‐tag (FLAG)‐LIFR (including S1044E and S1044A mutant)‐associated complexes in PCa cells that were pulled down with an anti‐Flag antibody were boiled at 95°C for 10 min. FLAG‐LIFR‐associated proteins were separated from the complexes using sodium dodecyl sulphate‐polyacrylamide gel electrophoresis (SDS‐PAGE) and were processed as previously described^19^ by reductive alkylation, trypsin digestion and peptide extraction. The peptides were analysed by liquid chromatography mass spectrometry (LC‐MS) on a Q Exactive mass spectrometer (Thermo Fisher Scientific). The fold change and *p*‐value were calculated based on the signal intensities of peptides from the LIFR‐S1044E‐ and LIFR‐S1044A‐associated proteins.

### 2.6 Statistics

Mouse experiments were performed with 6–12 mice, and cell experiments were independently repeated three times. Unless otherwise noted, SEM indicates the error bar of the statistical data in the figures, and the results were analysed by the two‐tailed Student's *t* test. A Cox proportional hazards regression model was implemented using SPSS software. The statistical significance of the data was determined by multiple methods, including one‐way ANOVA, two‐way ANOVA, Student's *t* test, Pearson's correlation coefficient, Wilcoxon's signed‐rank test, chi‐square test, log‐rank test and Shapiro–Wilk test. For the statistical data in all figures, a *p*‐value ≤ 0.05 indicates significance.

## 3 RESULTS

### 3.1 Extracellular lysine acetylation is clinically associated with PCa progression

**FIGURE 1:**
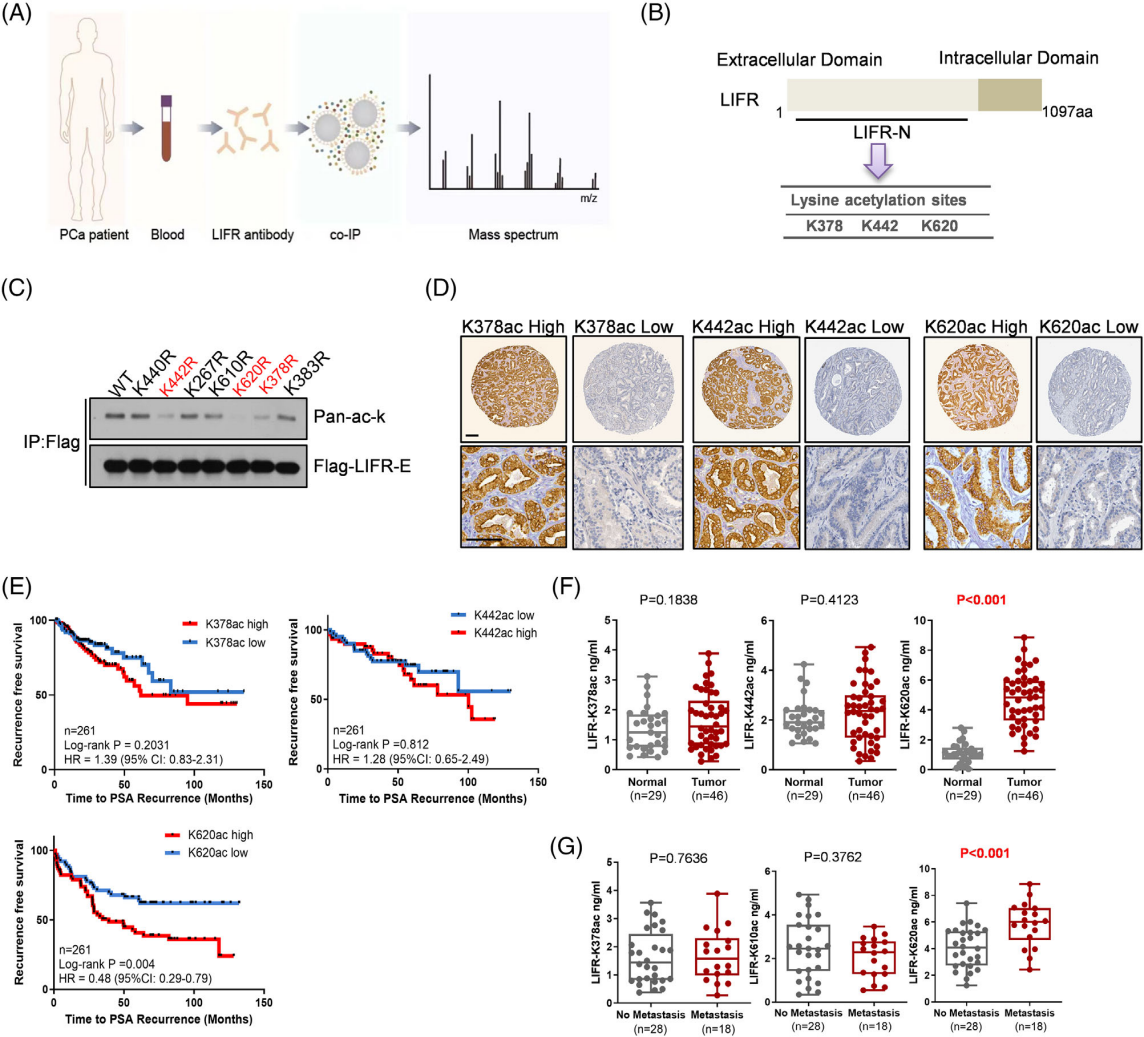
Extracellular lysine acetylation of LIFR is clinically associated with PCa progression. (A,B) Collection of blood samples and enrichment of LIFR for mass spectrometry. (C) Mutagenesis of extracellular lysine residues on LIFR to arginine residues for further identification of the primary acetylation site. (D) Representative IHC images using antibodies against LIFR‐AcK378, LIFR‐AcK442 or LIFR‐AcK620. Scale bars: 300 μm. (E) Kaplan–Meier plot of recurrence after radical prostatectomy based on the LIFR‐K378, LIFR‐K442 or LIFR‐K620 acetylation index in patients (*p*‐values by log‐rank test). Hazard ratio (HR). (F) The blood concentrations of LIFR‐AcK378, LIFR‐AcK442 or LIFR‐AcK620 in PCa patients and healthy controls and (G) in PCa patients with or without metastasis

The lack of clinically available accurate diagnostic markers results in delayed PCa diagnosis until it is in an advanced stage. To discover a new biomarker for PCa, we first collected blood samples from PCa patients and enriched the samples for soluble LIFR for peptide identification (Figure 1A, Table S1). As shown in Figure 1B, three lysine sites, K378, K442 and K620, were acetylated in blood samples from PCa patients upon extraction of the secondary mass spectra with high quality, we confirmed the acetylation modification at K378 and K620 (Figure 1B, Figure S1A). Next, we overexpressed the extracellular domain of LIFR in PC3 cells and tested the pan‐acetylation level after mutating individual lysine residues. Consistently, cells expressing the K620R mutant showed strong reductions in the total acetylation of extracellular LIFR, while cells expressing the K378R or K442 mutants showed a moderate decrease (Figure 1C). We prepared antibodies specifically against acetylated K378, K442, or K620. After we performed several routine affinity purification and enrichment steps, the specificity of the above antibodies was examined separately by WB (Figure S1B) and IHC (Figure S1C). Before the antibodies were used to evaluate the clinical relevance of the three acetylation modifications of the LIFR extracellular domain in PCa. IHC staining was performed with a TMA consisting of 261 PCa patient samples (Table S2, Figure 1D) from an Asian radical prostatectomy cohort described in a previous report.^20^ As shown in Figure 1E, patients with high LIFR‐K620 acetylation showed an increased risk of biochemical recurrence (BCR; *p* = 0.004; Figure 1E), but the other two sites did not correspond to an increased risk. Receiver operating characteristic (ROC) analysis and multivariate Cox analysis were conducted to clarify the diagnostic and prognostic efficacy of LIFR‐K620 acetylation in PCa, the results of which indicated that LIFR‐K620 acetylation is a potential biomarker that predicts PCa patient prognosis (Figure S1D, Tables S3 and S4).

We performed ELISA to determine the levels of these extracellular lysine acetylation modifications of LIFR using blood samples from healthy subjects and PCa patients, and we observed that acetylation of LIFR at K620, but not at the other two sites, was significantly upregulated in the blood of PCa patients (Figure 1F). Moreover, we further stratified the blood samples of PCa patients based on metastasis, as metastasis is lethal for most PCa patients. When PCa progressed to an advanced stage, LIFR‐K620 acetylation levels were even higher than those in patients with nonmetastatic PCa (Figure 1G). We also performed multivariate analysis to determine whether LIFR‐K620 acetylation can improve the diagnostic accuracy of Gleason scores and prostate specific antigen (PSA) levels by stratifying the BCR risk in patients from the Asian radical prostatectomy cohort (Table S2).

These results indicate the significant prognostic value of LIFR‐K620 acetylation in PCa and drove us to investigate the role of LIFR‐K620 acetylation in prostate tumourigenesis.

### 3.2 LIFR‐K620 acetylation is essential for LIFR homodimerization and subsequent LIFR‐S1044 phosphorylation

To transduce the IL6 or LIF signal, LIFR forms a heterodimer with GP130. However, an increasing number of investigations have suggested that LIFR and GP130 could play distinct roles in tumour occurrence and development.^21^, ^22^, ^23^, ^24^, ^25^, ^26^, ^27^, ^28^ Therefore, we suspected that LIFR and GP130 can separately execute their respective roles. A Co‐IP assay showed that LIFR and GP130 did not form a stable heterodimer (Figure S2A), and knockdown of LIFR or GP130 in PC3 cells affected the activation of different signalling pathways (Figure S2B). Consistently, ectopic expression of LIFR promoted AKT activation, while overexpression of GP130 induced constitutively higher Hippo signalling (Figure S2C).

**FIGURE 2:**
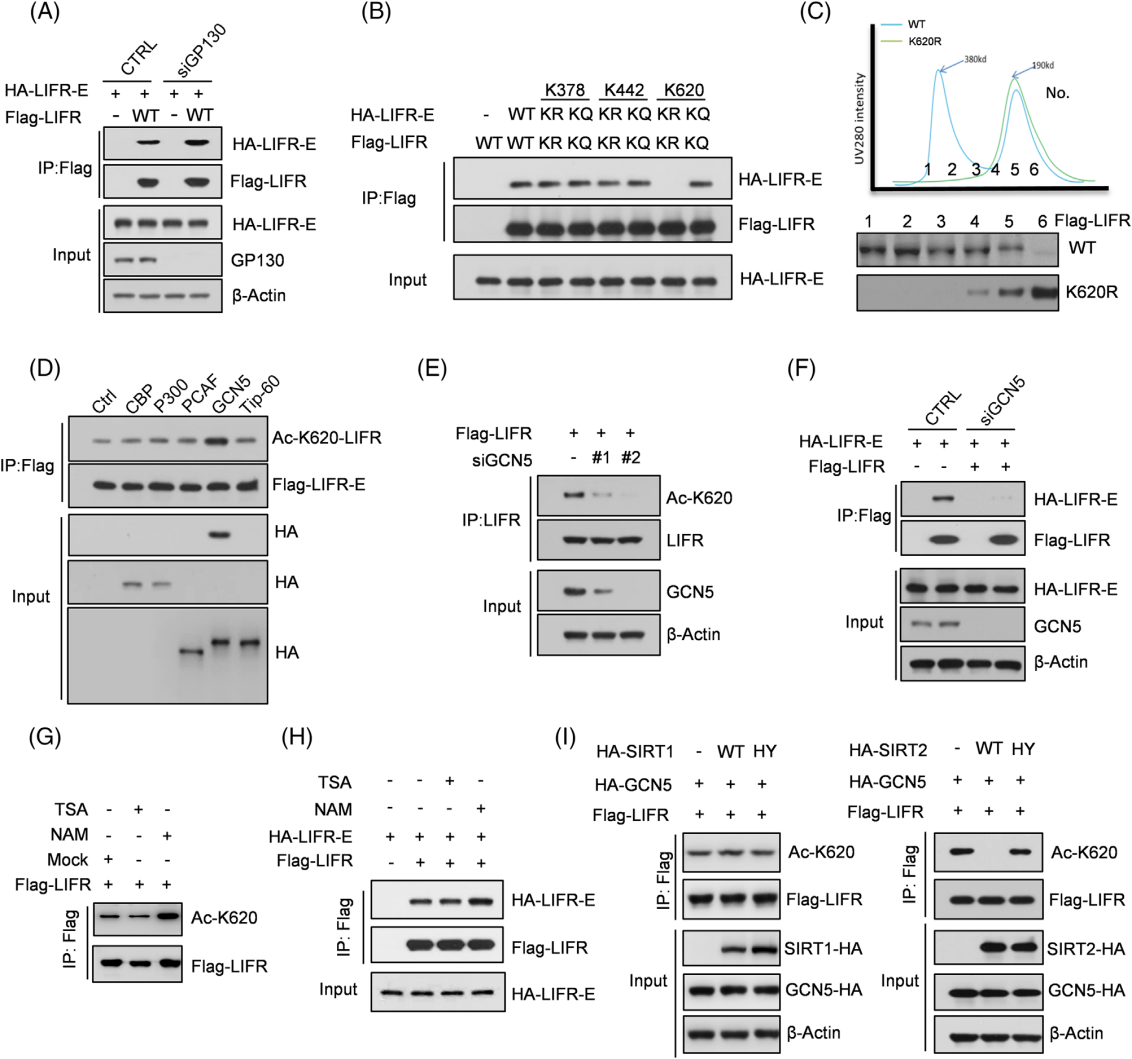
LIFR‐K620 acetylation is essential for LIFR homodimer formation and consequent LIFR‐S1044 phosphorylation. (A,B) Co‐IP was performed with FLAG‐M2 beads enriched with FLAG‐LIFR‐associated proteins. (C) Reverse‐phase high‐performance liquid chromatography (RP‐HPLC) of proteins bound to LIFR homodimers. (D,E) FLAG‐LIFR was stably expressed in PC3 cells transiently transfected with CBP, p300, PCAF, GCN5 or Tip60 (D) or with GCN5 depletion (E). IP was performed with FLAG‐M2 beads that enriched full‐length FLAG‐LIFR. (F) Myc‐LIFR‐extracellular and FLAG‐LIFR were cotransfected into PC3 cells with or without GCN5, and co‐IP was performed. (G) PC3 cells stably expressing FLAG‐LIFR were treated with TSA (1 μM) or NAM (50 μM) for 12 h, and IP was performed. (H) Myc‐LIFR‐extracellular and FLAG‐LIFR were cotransfected into PC3 cells treated with TSA or NAM for 12 h, and co‐IP was performed. (I) HA‐SIRT1 (WT or HY mutant) or SIRT2 (WT or HY mutant) was stably expressed, and IP was performed. HY mutants are catalytically inactive (H363Y and H187A, respectively)

To better understand the proteins regulated by LIFR and GP130, we used comparative proteomic profiling without labelling to quantify the differentially altered proteins. We observed that a minority of proteins were upregulated or downregulated in both the LIFR depletion and GP130 depletion groups (Figure S2D). In addition, our previous study showed that LIFR alone can form a homodimer depending on the intracellular lysine acetylation status to reinforce the LIF signal in embryonic stem cells.^19^ Thus, in PC3 cells, we depleted GP130, and the results of the co‐IP assays indicated that GP130 depletion did no impair the formation of the LIFR homodimer (Figure 2A), which completely disappeared upon loss of K620 acetylation (Figure 2B). Consistent with this, a gel filtration assay showed that a mimic acetylation mutant at LIFR‐K620 obtained by mutating lysine to glutamine (Q) could potentially form homodimers (Figure 2C).

There are five major acetyltransferases involved in lysine acetylation in mammalian cells: CBP, p300, PCAF, TIP60 and GCN5. We transiently overexpressed these acetyltransferases and found that GCN5 overexpression enhanced LIFR acetylation at K620, indicating that GCN5 acetylates LIFR at K620 in PCa cells (Figure 2D). Then, we transiently knocked down GCN5 and observed a dramatic decrease in the acetylation level at LIFR‐K620 (Figure 2E, Figure S3A,B) and a strong reduction in LIFR homodimerization (Figure 2F).

Acetylation is a kind of reversible modification that is carried out by deacetylases, including histone deacetylases (HDACs) and NAD‐dependent protein deacetylase sirtuin (SIRTs). Of note, extracellular lysine acetylation is reversed by secretory deacetylases. To identify which kinds of deacetylases contribute to LIFR‐K620 deacetylation, trichostatin A (TSA) and nicotinamide (NAM), which inhibit HDACs and SIRTs, respectively, were applied to cells, and we observed that after treatment with NAM, LIFR‐K620 acetylation increased, indicating that SIRTs were required for removal of the acetyl group at K620 (Figure 2G). Moreover, the homodimerization of LIFR was enhanced after NAM treatment (Figure 2H). We also detected SIRT1 and SIRT2 in the medium (Figure S3C). By transfecting wild‐type or dead‐enzyme mutants of SIRT1 or SIRT2 into tumour cells, we found that the loss of SIRT2 deacetylase activity resulted in consistent levels of LIFR‐K620 acetylation (Figure 2I). Moreover, GCN5 was observed to be overexpressed in PCa and positively correlated with PCa progression, while SIRT2 was negatively correlated with PCa progression, which is consistent with the vital role of LIFR‐K620 acetylation in PCa progression (Figure S3D–G). Of note, SIRT2 was previously reported to be secreted by PCa cells.^29^, ^30^

### 3.3 GCN5‐ and SIRT2‐mediated LIFR acetylation at K620 regulates its phosphorylation at S1044 and consequentially regulates AKT activity

**FIGURE 3:**
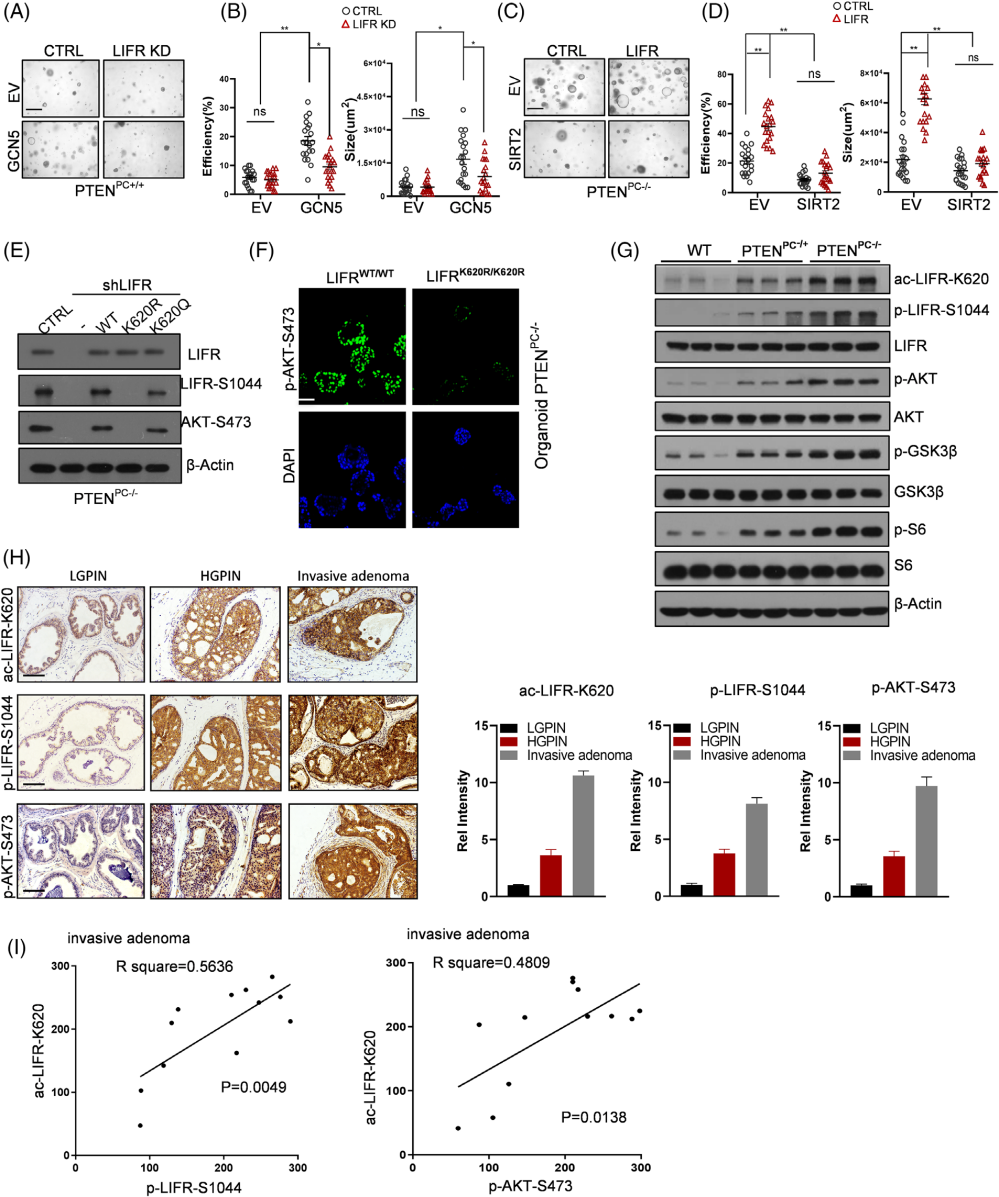
GCN5‐ and SIRT2‐mediated acetylation of LIFR at K620 promotes its phosphorylation at S1044 and consequent activation of AKT. (A,B) Representative images of the organoids are shown (A). The formation capacity of the organoids was analysed and calculated. Quantitation of the organoid size and efficiency is representative of three experiments (two‐tailed Student's *t* test) (B). (C,D) Representative images of the organoids are shown (C). The formation capacity of the organoids was analysed and quantified (D). (E) LIFR‐S1044 phosphorylation and AKT activation in organoids from PTEN‐null prostates after depletion of LIFR and rescued with WT, K620R or K620Q mutants were examined by WB using the indicated antibodies. (F) IF was performed in organoids from *Pten*‐null mice after depletion of LIFR and rescued with WT or K620R using the indicated antibodies. (G) WB analysis of phospho‐AKT, AKT, and their downstream targets (phospho‐S6 and phospho‐GSK3β) in anterior prostate tissues from 24‐week‐old mice as indicated. (H,I) PCa samples from PTEN^PC‐/+^ and PTEN^PC‐/–^ mice with low‐grade PIN, high‐grade PIN and invasive PIN were tested by IHC using the indicated antibodies against LIFR‐AcK620, phospho‐S1044 and activated AKT (*n* = 6) (H). Kendall's tau‐beta was used to test for correlations in the IHC results (I). Scale bars: 800 μm (A and C), 50 μm (F and H). ^**^ *p* < 0.01

Next, an organoid formation experiment was utilized to assess the functional regulation of LIFR‐K620 acetylation in vivo. We isolated luminal cells from the prostate of TMPRSS2^CreERT2‐IRES‐GFP^; *Pten* ^fl/fl^ mice.^31^, ^32^ GCN5 enhanced the organoid formation capacity, which was blocked by LIFR knockdown, indicating that LIFR is the main downstream target of GCN5 in PCa. Furthermore, SIRT2 overexpression reduced the organoid formation capacity and blocked the activity induced by LIFR overexpression, which is consistent with the reported tumour suppressor role of SIRT2^33^ and SIRT2‐mediated effects on LIFR function (Figure 3A–D).

Once we mimicked the constitutive deacetylation of LIFR‐K620, S1044 phosphorylation and AKT‐S473 phosphorylation were heavily reduced (Figure 3E,F). WB further showed that upregulated LIFR‐K620 acetylation, LIFR‐S1044 phosphorylation and AKT‐S473 phosphorylation were followed by PCa progression and acted on the AKT signalling pathway, as shown by changes in the downstream effectors of PI3K/AKT signalling, phospho‐S6 and phospho‐GSK3β (Figure 3G).

Next, we examined LIFR‐K620 acetylation, LIFR‐S1044 phosphorylation and AKT‐S473 phosphorylation using samples from patients with low‐grade prostatic intraepithelial neoplasia (low‐GPIN), high‐GPIN, or invasive adenocarcinoma by IHC. As shown in Figure 3H, the levels of LIFR‐K620 acetylation, LIFR‐S1044 phosphorylation and AKT‐S473 phosphorylation increased as the PCa disease stage advanced (Figure 3H). There was a significant positive correlation between LIFR‐K620 acetylation and LIFR‐S1044 phosphorylation and between LIFR‐K620 acetylation and AKT‐S473 phosphorylation (Figure 3I). Hence, LIFR‐K620 acetylation reinforced its homodimerization and consequentially promoted its downstream signalling.

### 3.4 LIFR‐K620 mutation inhibits the progression of PTEN‐deleted tumours in mice

**FIGURE 4:**
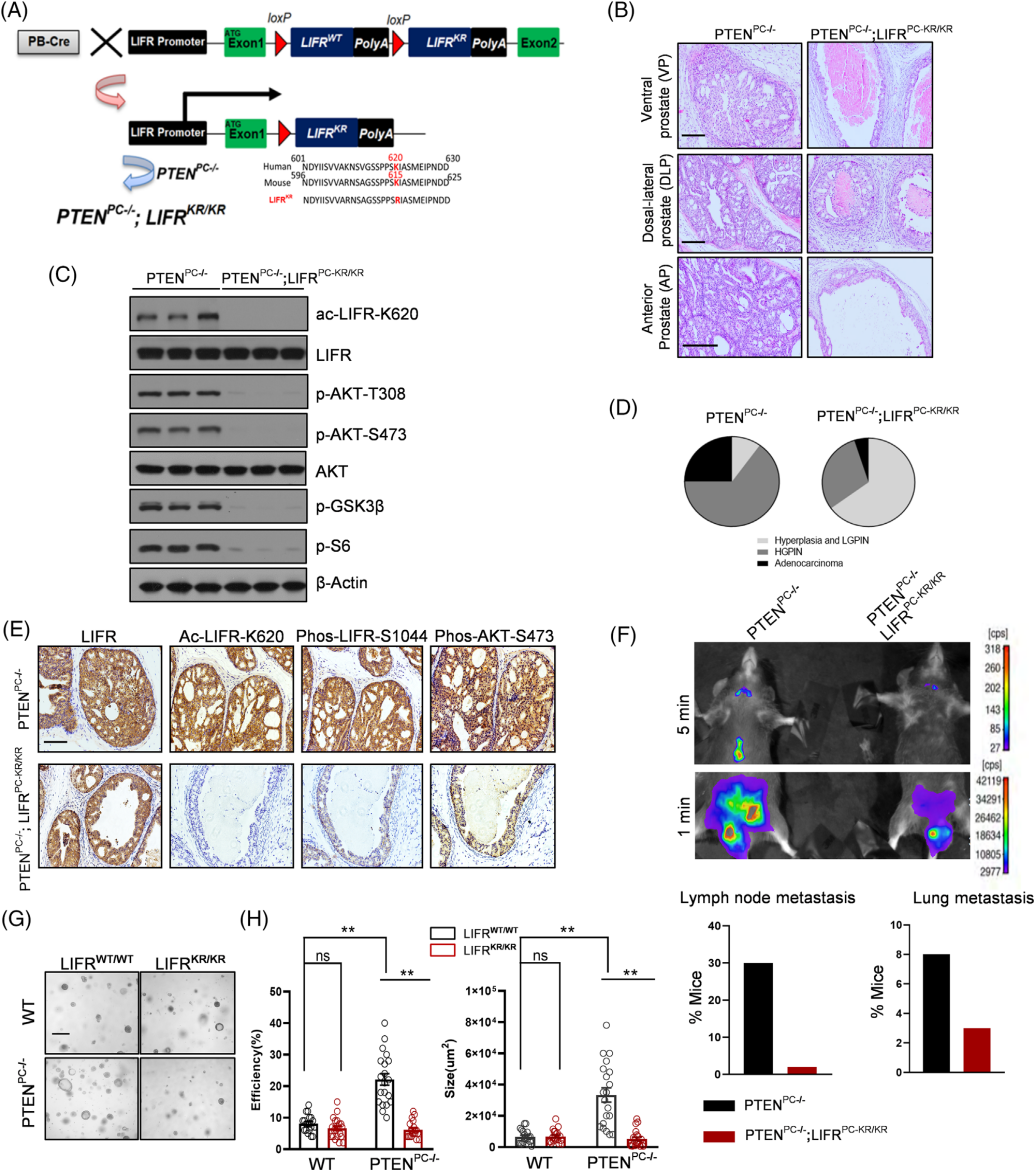
LIFR‐K620 acetylation is required for PCa progression. (A) Scheme of the conditional LIFR‐KR mutation in the PTEN^PC‐/–^ mouse model. The mouse LIFR‐K615 site is analogous to human LIFR‐K620. (B) H&E‐stained sections of representative anterior prostate (AP), dorsal‐lateral prostate (DLP), and ventral prostate (VP) of 6‐month‐old mice. (C) WB analysis of the indicated proteins in AP lysates from PTEN^PC–/–^ and PTEN^PC–/–^ LIFR^PC‐KR/KR^ mice. (D) Pie graphs show the results of tumour progression in the indicated mice at 6 months of age. HGPIN, high‐grade PIN; LGPIN, low‐grade PIN. (E) IHC analyses of LIFR, ac‐LIFR‐K620, phospho‐LIFR‐S1044 and phospho‐AKT‐S473 levels in prostate tumours from the indicated mice at 6 months of age. (F) Representative luminescence images of the indicated mice at 6 months of age. Quantification results of distal metastasis for the examined animals are shown in the bottom panel (*n* > 12). Fisher's exact test was used to determine statistical significance. (G,H) Representative images of organoids from the indicated mice (age of 24 weeks) (G). The formation capacity of the organoids was analysed and quantified. Quantitation of the organoid size and efficiency is representative of three experiments (two‐tailed Student's *t* test) (H). **p < 0.01, *p < 0.05. Scale bars: 50 μm (A and B), 800 μm (G)

Mouse LIFR‐K615 is the analogue of human LIFR‐K620. Therefore, we generated LIFR‐KR mutation transgene mice by specifically mutating mouse LIFR K615 to arginine (R) in the prostate epithelium in vivo (Figure 4A). Histopathological examination of prostates indicated that LIFR‐K615 acetylation was not physiologically required for prostate homeostasis or development (Figure S4A). Further immunostaining for cytokeratin 8 (CK8) and α‐smooth muscle actin (α‐SMA) supported this result (Figure S4A). Overall, under steady‐state conditions, no obvious differences between control and LIFR^PC‐KR/KR^ mice were observed.

The prostate‐specific Pten‐depleted mouse model is considered an outstanding in vivo model of PCa with histopathological features similar to those of human disease, which recapitulates the progression of prostate tumours.^14^, ^34^ We sought to determine whether mouse LIFR‐K615 acetylation is essential for PTEN‐deleted tumour progression. We crossed *Pten* ^fl/fl^ mice with LIFR^PC‐KR/KR^ mice to establish mice with the LIFR‐K615 mutation to arginine on a PTEN‐deleted background (hereafter referred to as PTEN^PC–/–^ LIFR^PC‐KR/KR^ mice; Figure 4A). The histopathological analysis of the whole GEM cohort revealed that the LIFR‐KR mutation impaired the progression of PCa in vivo. As indicated in Figure 4B, D, the prostate of 4‐month‐old PTEN^PC–/–^ mice mostly progressed to adenocarcinoma or high‐grade PIN (HGPIN), with less locally invasive carcinoma. By comparison, the prostate of PTEN^PC–/–^ LIFR^PC‐KR/KR^ mice conversely developed at the low PIN stage with fewer lesions and markedly impaired tumour progression even after 4 months (Figure 4B, D). Consistently, the LIFR‐KR mutation significantly reduced the level of LIFR‐S1044 phosphorylation and AKT signalling pathway activation in vivo (Figure 4C,E).

To monitor tumour growth and metastasis in vivo, Rosa26‐LSL‐luciferase reporter mice were crossed with LIFR mutant mice to allow for imaging studies. As shown in Figure 4F, PTEN^PC–/–^ LIFR^PC‐KR/KR^ mice showed a much weaker bioluminescence intensity, which corresponds to impaired tumour growth in vivo. Moreover, tumour cells in control mice metastasized to distal organs (such as lungs and lymph nodes), while those in LIFR‐KR mutant mice displayed lower penetrance of bioluminescence intensity in the distal organs (Figure 4F). Furthermore, we performed organoid assays to determine whether LIFR‐K620 acetylation functions in PCa progression in a PTEN‐dependent manner. Luminal cells from prostates of 3‐month‐old WT or LIFR‐KR mice with intact or deficient PTEN were isolated. LIFR‐KR impaired the organoid formation capacity in PTEN‐null mice but not in PTEN‐intact mice (Figure 4G,H), indicating that the loss of mouse LIFR‐K615 acetylation prevented the progression of tumours with PTEN deletion; this outcome was reinforced by further histopathological analyses. The number of Ki‐67‐positive cells was reduced significantly in the PTEN^PC–/–^ LIFR^PC‐KR/KR^ group compared with the PTEN^PC–/–^ group, revealing that tumour cells bearing the LIFR‐KR mutation inherited disadvantageous growth traits (Figure S4B). Moreover, some invasive tumour cells in PTEN‐null mice underwent epithelial‐mesenchymal transition (EMT), as indicated by reduced expression of the epithelial marker E‐cadherin and increased expression of the mesenchymal marker vimentin, which revealed that tumour cells bearing LIFR‐KR mutations inherited disadvantageous metastasis traits (Figure S4C).

Collectively, these GEM model results highlight that LIFR‐K620 acetylation cooperates with PTEN loss to promote PCa progression.

### 3.5 LIFR‐S1044 phosphorylation activating the AKT pathway is dependent on PDPK1 recruitment and PTEN loss

**FIGURE 5:**
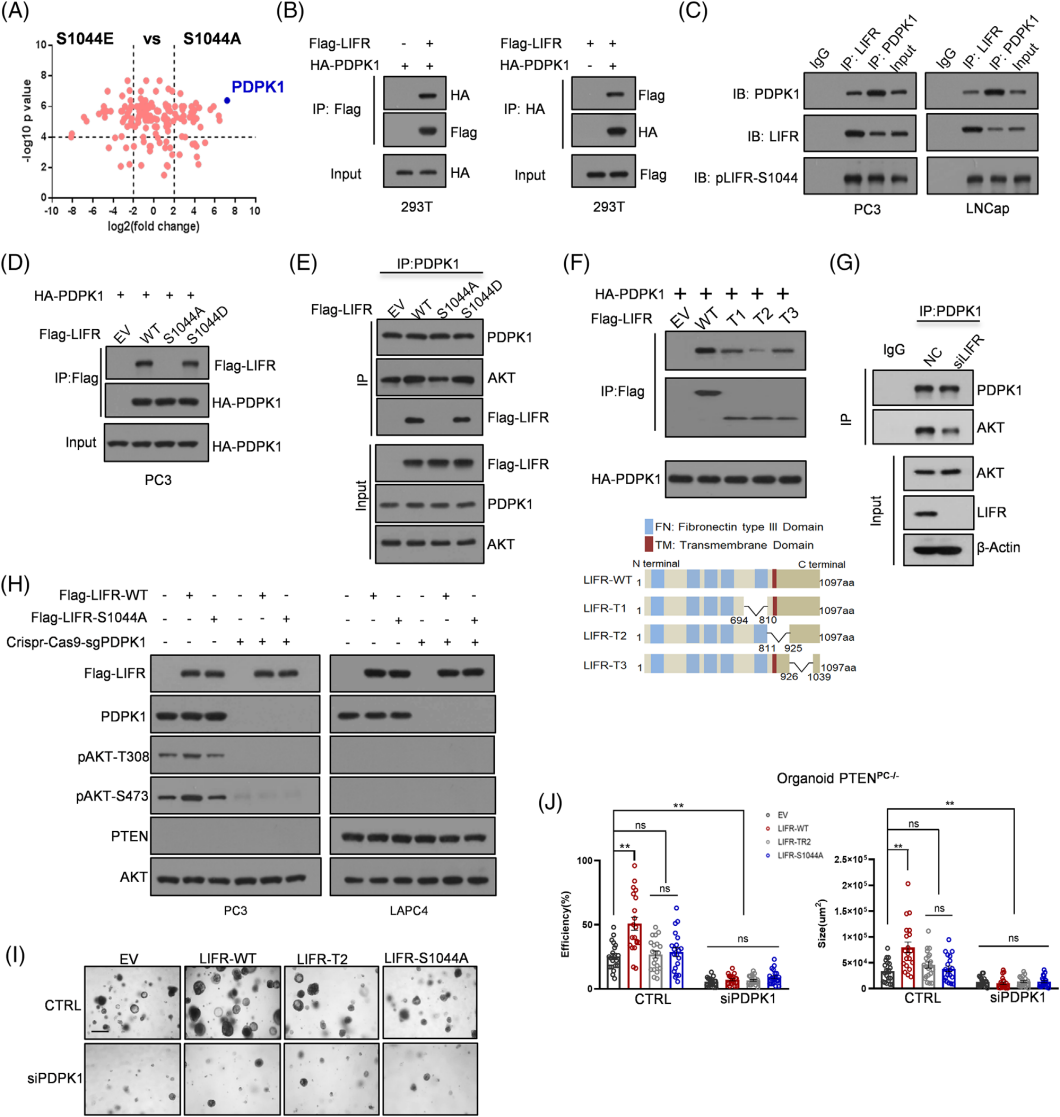
LIFR‐S1044 phosphorylation‐mediated activation of AKT is dependent on PDPK1 recruitment and PTEN loss. (A) Mass spectrometry analyses of LIFR‐associated proteins in PC3 cells stably expressing FLAG‐LIFR (S1044E or S1044A). (B) Co‐IP was performed to assess the interaction between FLAG‐LIFR and HA‐PDPK1. (C) The endogenous interaction between LIFR and PDPK1 in PC3 and LNCap cells was examined using the indicated antibodies. (D,E) The forward (D) and reverse (E) interactions between FLAG‐LIFR (WT, S1044A or S1044E) and HA‐PDPK1 were evaluated using co‐IP. (F) Mapping of the region in the LIFR intracellular domain required for PDPK1 recruitment. (G) PDPK1 was knocked down with a specific small interfering RNA (siRNA). Then, FLAG‐LIFR (WT or S1044A) was transfected into the indicated cells. The indicated antibodies were used to measure AKT activation. (H) The endogenous interaction of PDPK1 and AKT was examined in PC3 cells with or without LIFR. (I,J) Representative images of PTEN‐null prostate organoids overexpressing LIFR (including WT, TR2 or S1044A mutant) with or without PDPK1 knockdown (I). The formation capacity of the organoids was analysed and calculated (J). The *p*‐values were calculated by two‐tailed Student's *t* test. **p < 0.01. ns, not statistically significant. Scale bar, 800 μm

To explore the downstream signalling regulated by LIFR, FLAG‐LIFR‐S1044E and FLAG‐LIFR‐S1044A were stably expressed in PC3 cells. The associated proteins immunoprecipitated with LIFR were identified by LC‐MS. Compared to FLAG‐LIFR‐S1044A, FLAG‐LIFR‐S1044E had 147 proteins with an increased interaction and 103 proteins with a decreased interaction. Among the proteins with an increased interaction with FLAG‐LIFR‐S1044E, 1,3‐phosphoinositide‐dependent protein kinase 1 (PDPK1) had an over four‐fold increased interaction with FLAG‐LIFR‐S1044E (*p* < 0.0001), indicating that PDPK1 is a strong candidate that facilitates the signal transduction of LIFR to AKT (Figure 5A, Figure S5A).

PDPK1 is responsible for the phosphorylation of AKT‐T308, which induces the conformational change of AKT to expose S473; consequently, S473 is either autophosphorylated or phosphorylated by mTORC2.^35^ We confirmed this interaction in HEK293T cells via forward and reverse Co‐IP assays with FLAG‐LIFR and HA‐PDPK1 (Figure 5B). We then examined this interaction with endogenous LIFR and PDPK1 in PC3 and LNCap cells, both of which show strong levels of LIFR‐S1044 phosphorylation (Figure 5C). We observed that mimicking the phosphorylation of LIFR at S1044 reinforced the interaction between LIFR and PDPK1 (Figure 5D,E). LIFR belongs to the family of type I cytokine receptors that usually form heterodimers with GP130.^36^ However, we found that LIFR could recruit PDPK1 even in the absence of GP130, indicating that LIFR‐mediated recruitment of PDPK1 is independent of GP130 (Figure S5B).

We further determined the region of LIFR required for recruiting PDPK1 by constructing LIFR truncation mutants, as shown in Figure 5F, and clarified the correlation between LIFR‐S1044 phosphorylation and PDPK1‐induced AKT activation (Figure 5G). We observed that LIFR promoted AKT signalling in PCa cells but not under the conditions of PDPK1 blockade, PTEN WT expression, or LIFR‐S1044 mutant expression, which indicated that the signal transduction of LIFR in PCa was dependent on PDPK1 recruitment and a lack of functional PTEN (Figure 5H). As shown in Figure 5I, overexpression of WT LIFR in *Pten* ^fl/fl^ mouse organoids promoted organoid formation, whereas LIFR mutants, such as the LIFR‐S1044A and LIFR‐TR2 deletion mutants, did not promote organoid formation. These results suggested that LIFR‐S1044 phosphorylation and the TR2 domain are essential for the function and signal transduction mediated by LIFR‐K620 acetylation in PCa progression (Figure 5H,I). Taken together, these data indicated that the signal transduction of LIFR‐K620 acetylation to AKT was dependent on PDPK1 recruitment and PTEN loss and was not affected by GP130.

### 3.6 LIFR‐K620 acetylation promotes PCa progression via LIFR‐S1044 phosphorylation‐dependent activation of AKT

**FIGURE 6:**
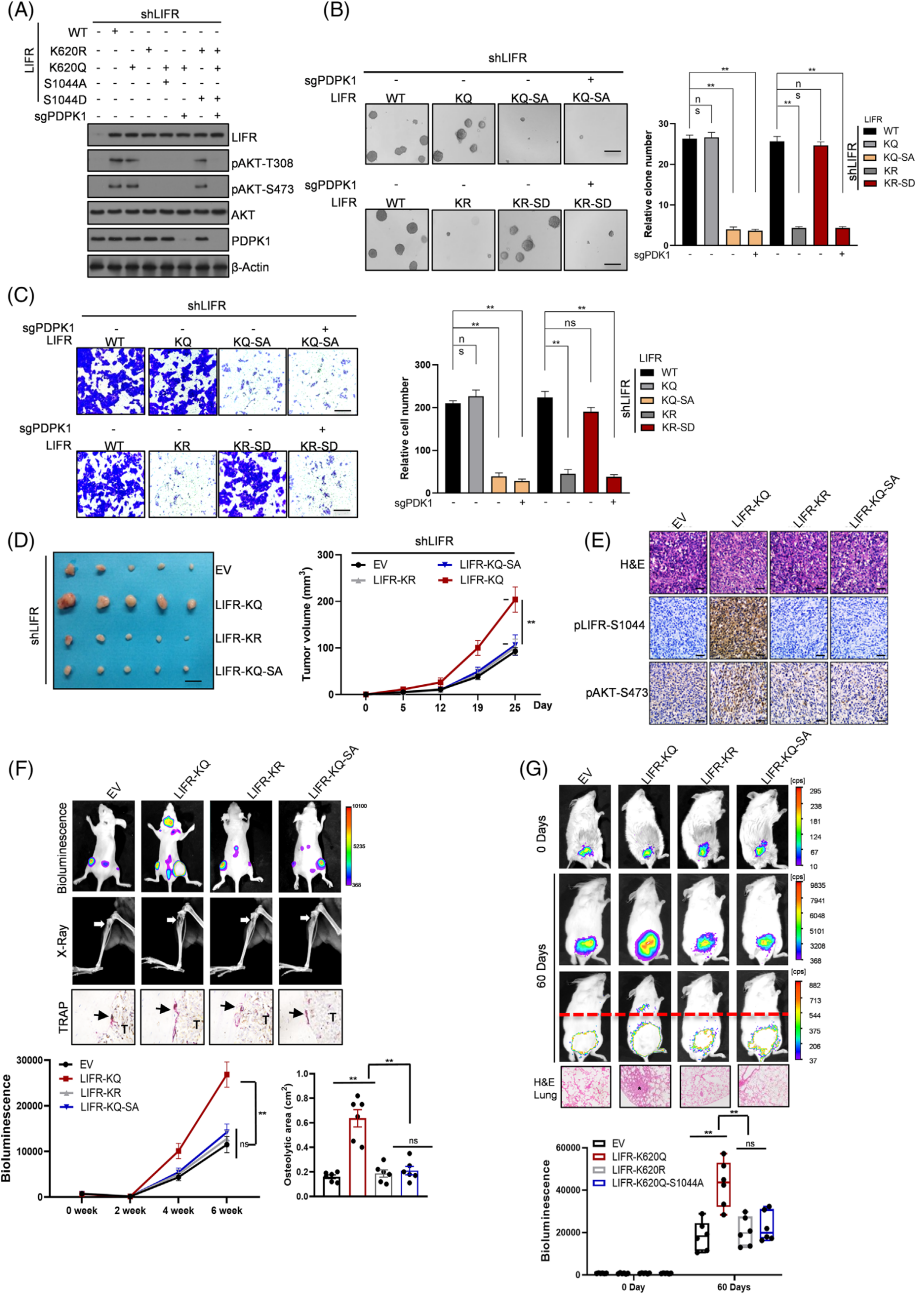
LIFR‐K620 acetylation‐driven PCa progression is dependent on LIFR‐S1044 phosphorylation‐mediated activation of AKT. (A) WB analysis of LIFR, phospho‐AKT‐S473, phospho‐AKT‐T308, total AKT and PDPK1 expression levels in the indicated samples. (B,C) Soft agar colony formation (B) and transwell (C) images of PC3 cells overexpressing WT LIFR or LIFR mutants with or without PDPK1 knockdown. (D) Measurement of subcutaneous tumour growth of PC3 cells overexpressing WT LIFR or LIFR mutants (*n* = 6, two‐way ANOVA followed by Tukey's multiple comparisons test). (E) H&E‐, phospho‐LIFR‐S1044‐, and phospho‐AKT‐S473‐stained images of subcutaneous tumour sections from the mice as indicated. (F) The top panel shows representative BLI from each group (*n* = 9 per group). The second top panel shows the representative X‐ray images of bone metastasis, and sizes of the osteolytic areas are quantified in the bottom right panel. The third top panel shows TRAP‐stained images of bone sections from the mice as indicated. T, tumour cell; arrow, TRAP‐positive stained cells. One‐way ANOVA followed by Tukey's multiple comparisons test. (G) Representative images and quantification of subcutaneous organoids as indicated (*n* = 6). The quantification of the volume of the subcutaneous organoids is presented as the mean ± SEM on the bottom panel and was analysed using two‐tailed Student's *t* test. Scale bars: 1 mm (B), 100 μm (C), 1 cm (D), 50 μm (E). **p < 0.01

We next examined whether LIFR‐S1044 phosphorylation is essential for the augmentation of AKT activation induced by LIFR‐K620 acetylation. As shown in Figure 6A, the expression of LIFR‐WT and LIFR‐K620Q rescued AKT activation in LIFR‐depleted PC3 cells but not K620R cells. In addition, K620Q‐S1044A could not rescue AKT activation, but K620R‐S1044D could, indicating that S1044 phosphorylation was directly downstream of K620 acetylation. In PDPK1‐depleted PC3 cells, LIFR functioned in an AKT activation‐dependent manner, and LIFR‐K620Q and K620Q‐S1044D could no longer activate AKT (Figure 6A). In line with the observation of cell signalling detected by WB, the cell growth, invasion and migration as assessed by soft agar and migration experiments were correlated with the regulation of phosphorylated AKT by LIFR mutants in LIFR‐depleted or PDPK1‐depleted PC3 cells (Figure 6B,C). As PC3 cells do not express androgen receptor (AR) and their proliferation is independent of androgen, we utilized another PCa cell line, C4‐2B, which are hormone‐resistant and have positive AR expression, to further verify our findings. C4‐2B cells were subjected to LIFR knockdown and then rescued with WT or mutant LIFR (K620R and K620Q), respectively. Consistent with the results in PC3 cells, WT and K620Q‐LIFR expression in C4‐2B cells abolished the loss of AKT activation induced by knockdown of WT LIFR but not of LIFR‐K620R. Furthermore, we detected the growth and metastasis of these constructed C4‐2B cells. K620‐LIFR acetylation promoted the growth and metastasis of PCa cells under AR‐positive or AR‐negative conditions (Figure S6A–C). Next, we utilized a xenograft model and an intracardiac injection assay to verify the in vitro results. Tumour volume examination and histopathological analyses indicated that PCa growth promoted by LIFR‐K620 acetylation and homodimer formation was directly dependent on downstream LIFR‐S1044 phosphorylation and activation (Figure 6D,E). Consistently, BLI showed that ectopic expression of LIFR‐K620Q in tumour cells resulted in the preferential metastasis to the bone 4 weeks after injection, whereas the control cells and cells expressing the other two mutant LIFR proteins (K620R and K620Q‐S1044A) displayed significantly fewer luminescent signals (Figure 6F, top). Two months later, mice injected with LIFR‐K620Q presented severe bone lesions and a prominent increase in osteolysis (Figure 6F, middle, X‐ray). In sharp contrast, there was little difference in tumour metastasis within the bone between the control and other two mutant groups (Figure 6F, top and middle); histopathological examination was used to further confirm this observation (Figure 6F, bottom). Moreover, organoids were orthotopically transplanted into recipient mice to further validate the results. In the organoid transplantation assay, organoids overexpressing LIFR‐K620Q increased the growth and metastasis of tumours transformed from PTEN‐deleted cells, but those expressing the other two mutants (K620R and K620Q‐S1044A) did not (Figure 6G). For further confirmation of the regulation in human samples, we used organoids derived from PCa patients with PTEN WT or PTEN‐null PCa and detected the levels of K620‐LIFR acetylation, S1044‐LIFR phosphorylation, AKT activation and its downstream signalling by Western blot (Figure S6D). These results confirmed the importance of K620‐LIFR in PI3K‐AKT signalling in PCa patients. Together, these results suggest that LIFR‐K620 acetylation promotes PCa progression directly via LIFR‐S1044 phosphorylation in an AKT‐dependent manner.

### 3.7 AKT promotes LIFR activation by upregulating GCN5 protein levels to acetylate LIFR‐K620

**FIGURE 7:**
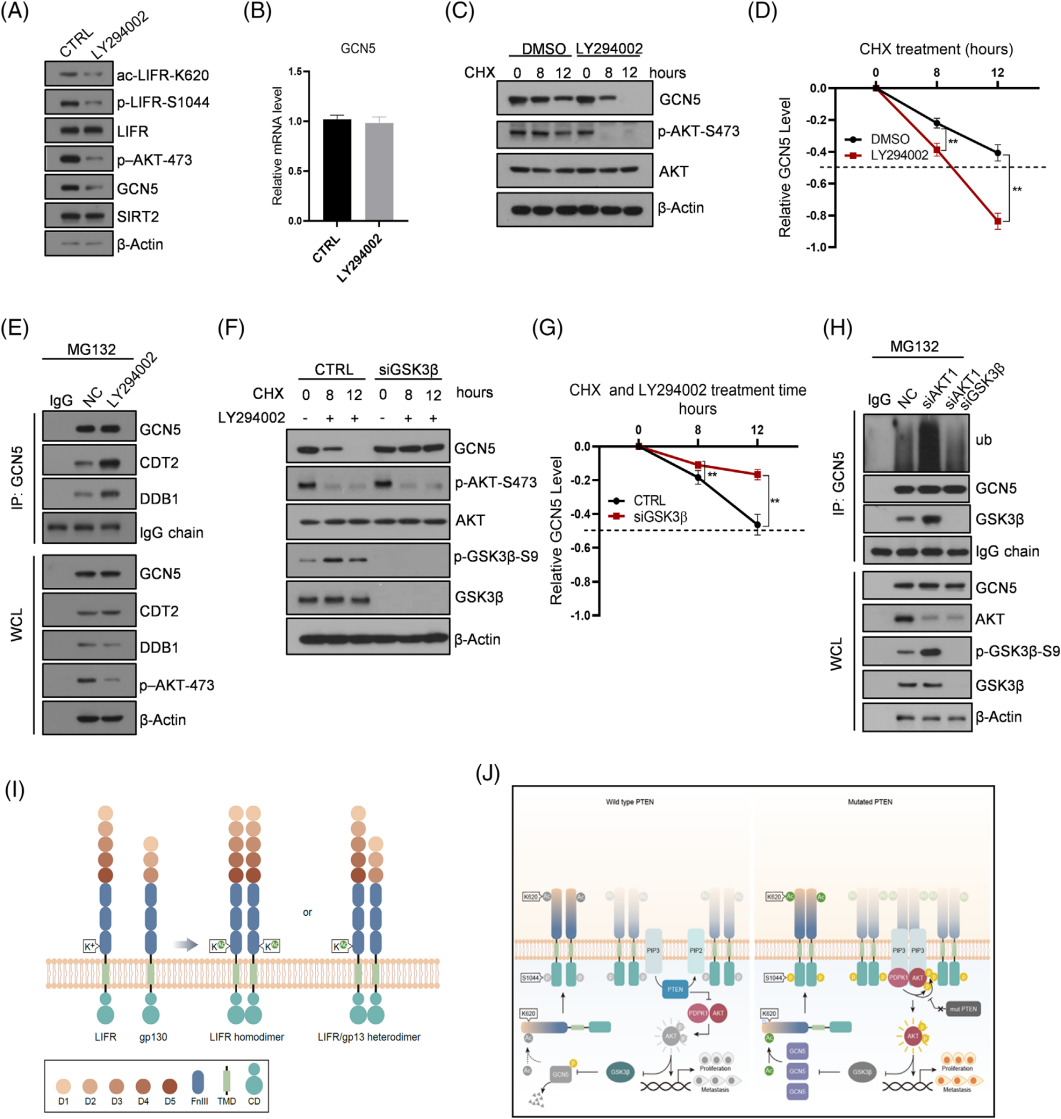
AKT promotes LIFR activation by upregulating GCN5 protein levels, which acetylate LIFR‐K620. (A) PC3 cells were treated with the AKT inhibitor LY294002 (25 μM) for 12 h, and the indicated antibodies were used to examine the signals. (B) qRT‐PCR was used to examine GCN5 mRNA levels in PC3 cells with or without LY294002 (25 μM) treatment for 12 h. (C,D) GCN5 protein was examined by WB in PC3 cells with or without LY294002 (25 μM) treatment for 12 h. Cycloheximide (CHX, 200 nM)‐treated PC3 cells for 0, 8 and 12 h (C). The relative GCN5 protein level was calculated and analysed by two‐tailed Student's *t* test (D). (E) The endogenous interaction between GCN5 and CDT2 and DDB1 was determined using an antibody against GCN5. (F,G) GSK3β was depleted in PC3 cells treated with LY294002 (25 μM) and CHX (200 nM) (F). The relative GCN5 protein level was calculated and analysed by two‐tailed Student's *t* test (G). (H) PC3 cells were depleted of AKT1 either alone or in conjunction with GSK3β before they were treated with MG132 for 12 h. An antibody against GCN5 was used to enrich GCN5‐associated proteins. The indicated antibodies were used to examine the ubiquitination of GCN5 and the GCN5/GSK3β interaction. (I,J) K620 acetylation mediated the formation of the LIFR homodimer (I). Schematic of the proposed mechanism identified in this study (J). **p < 0.01

As AKT is a major kinase required for PCa progression, we tested whether the AKT activity contributes to the GCN5/LIFR signalling axis. Treatment of PC3 cells with the AKT inhibitor LY294002 decreased the protein expression of GCN5, acetylation at LIFR‐K620 and phosphorylation at LIFR‐S1044, primarily indicating that the AKT kinase activity could play an important role in the GCN5/LIFR axis activity by maintaining GCN5 at the protein level but not the mRNA level (Figure 7A,B and Figure S6E). After co‐treatment with cycloheximide (CHX), an inhibitor of protein synthesis, inhibition of the AKT activity accelerated the reduction in GCN5 protein levels, as shown by Western blot and the corresponding statistical analysis (Figure 7C,D). Previous studies have reported that GCN5 protein stability is regulated by the CRL4^Cdt2^ E3 ubiquitin ligase, which is composed of the CUL4A/DDB1/CDT2 complex,^37^ and that increased interactions between GCN5 and the CRL4^Cdt2^ E3 ubiquitin ligase contributes to the downregulation of GCN5 via proteasome‐mediated degradation.^38^ In our study, we first showed that GCN5 could associate with CRL4^Cdt2^ E3 ubiquitin ligase in PCa cells; moreover, the inhibition of the AKT activity reinforced this association and increased GCN5 degradation (Figure 7E). GCN5 has no AKT substrate‐like sequence motifs but harbours several GSK3β consensus sequence motifs, and GSK3β is one of the main substrates of AKT (Figure S6F). Thus, we tested whether AKT regulated GCN5 stability via GSK3β. Upon GSK3β depletion, cells exhibited more stable GCN5 protein levels, even upon AKT inhibition, suggesting that the LIFR/PDPK1/AKT axis transduces signals from AKT to GSK3β/GCN5 (Figure 7F,G). To further confirm the existence of the AKT/GSK3β/GCN5 axis, we depleted AKT either alone or in conjunction with GSK3β. When both AKT and GSK3β were depleted, the ubiquitination of GCN5 decreased to a level similar to that in the control cells, indicating that GSK3β activity negatively regulates GCN5 protein stability through the ubiquitin–proteasome pathway (Figure 7H).

Together, these results showed that the upregulation of LIFR in the prostate epithelium is dependent on AKT activation and is required for PTEN‐null tumour progression. Mechanistically, AKT maintains GCN5 protein stability by antagonizing GCN5 degradation via GSK3β‐mediated CRL4^Cdt2^ E3 ligase activity. Next, increased GCN5 expression acetylates LIFR at K620, resulting in the further AKT activation and PCa progression with a positive feedback loop. These results harbour significant clinical relevance to PCa malignancy due to the high frequency of PTEN mutations and hyperactivated PI3K/AKT signalling in PCa.

## 4 DISCUSSION

PCa is common disease and remains the leading cause of cancer‐related death in males. The treatment of patients with metastatic PCa is a major global health care challenge. When metastatic PCa is diagnosed, androgen deprivation therapy (ADT) is adopted as the initial line of treatment; however, ADT is a palliative rather than curative treatment for advanced PCa patients, who eventually develop metastatic castration‐resistant PCa, which has limited efficacious treatment options currently available.^39^, ^40^, ^41^, ^42^ Thus, there is an unmet need for improving the performance of screening and monitoring advanced PCa.

Notably, posttranslational modifications of membrane receptors often transduce ligand signals from the pathogenetic microenvironment to activate downstream signalling pathways within the cells and thereby promote disease progression. Moreover, many truncated receptors with only extracellular domains arising from the shearing of full‐length receptors or production of alternatively spliced transcripts are soluble in the blood and highly correlated with disease progression. Thus, posttranslational modifications of soluble receptors in the blood may be viable biomarkers of disease.^9^, ^43^ At least three LIFR transcripts are transcribed from the gene. The shortest form (3 kb) is generated by the introduction of a novel stop codon to truncate the mRNA, resulting in the soluble form of LIFR that lacks transmembrane and intracellular domains and comprises only the extracellular domain.^44^ In our previous work, LIFR‐S1044 phosphorylation activated the AKT pathway to promote PCa progression.^20^ Given that the soluble form of LIFR exists in the circulatory system,^45^ we suspected that LIFR could be exploited to monitor PCa progression by examining blood samples. Additionally, acetylated proteins mark disease progression; for example, acetylated tau is a novel pathological signature in Alzheimer's disease,^46^ and acetylated tau in the blood could be applied clinically to examine patients with traumatic brain injury.^11^ Further analysis of clinical samples showed that LIFR‐K620 acetylation could be a biomarker for monitoring PCa progression.

Although LIFR‐K620 acetylation shows clinical significance, how LIFR forms a homodimer to activate its downstream signalling pathway in cancer remains unclear. Here, we found that the K620 acetylation‐mediated formation of the LIFR homodimer drives AKT signalling independent of GP130 in PCa cells, although GP130 can act as a coreceptor that can bind interleukin‐11 (IL‐11), leukaemia inhibitory factor (LIF), oncostatin M (OSM), ciliary neurotrophic factor (CNTF), and cardiotrophin‐1 (CT‐1).^47^ Hence, the genes regulated by GP130 should almost overlap with those mediated by LIFR. However, in this study, LIFR and GP130 activate different downstream pathways in PCa, indicating that the GP130/LIFR heterodimer may not represent the main avenue by which LIFR transduces extracellular signals.

To identify the acetyltransferase required for LIFR‐K620 acetylation, we transiently depleted or overexpressed some acetyltransferases and preliminarily confirmed that GCN5 is required for LIFR‐K620 acetylation. GCN5 is mainly localized in the nuclei; however, one report showed that the substrates of GCN5 are distributed across numerous cellular compartments with multiple functions.^48^ Lysine acetylation is a reversible chemical modification. To identify the deacetylase that contributes to the deacetylation of LIFR‐K620, we first applied deacetylase inhibitors and then investigated whether the deacetylases were secreted in the extracellular space, which is required for the deacetylation reactions. Additionally, we excluded SIRT4/5, which has weak deacetylase activity.^49^, ^50^ Although both SIRT1 and SIRT2 were secreted into the extracellular space, only SIRT2 deacetylated LIFR‐K620 acetylation, indicating that the tumour microenvironment of PCa has certain characteristics and functions.

PTEN mutations and excess PI3K/AKT signalling are generally considered to be pathologically related to PCa because approximately 30% of primary PCa and nearly 70% of metastatic PCa are mutated at the genome locus of the PTEN gene, demonstrating that the PTEN and PI3K/AKT signalling pathways play a key role in the progression of PCa.^34^, ^51^ Similarly, GEM models demonstrated a vital role of PTEN‐AKT signalling in PCa.^14^ Consistent with this point, we observed that LIFR‐K620 acetylation‐mediated activation of AKT signalling depends mainly on PTEN deletion, suggesting that inhibiting LIFR‐K620 acetylation is a potential strategy to treat advanced PCa with PTEN loss.

GSK3β is a promiscuous kinase and one of the first identified substrates of the oncogenic kinase AKT, and phosphorylation at S9 inhibits GSK3β activity owing to the formation of an autoinhibitory pseudosubstrate sequence.^52^, ^53^ In addition, GSK3β has been shown to phosphorylate several upstream and downstream components of the PI3K/AKT/mTOR signalling network, including RICTOR,^54^ TSC1 and 2,^55^ insulin receptor substrate (IRS)1 and IRS2,^56^ with the potential to apply feedback control within the network. Here, we found that GSK3β mediates GCN5 binding with the CRL4‐CDT2 E3 ligase complex and its subsequent degradation by the proteasome pathway. This degradation of GCN5 is inhibited by AKT activation, leading to increased GCN5 protein levels and subsequently higher levels of LIFR‐K620 acetylation in PTEN‐null tumour cells, eventually forming a positive feedback loop to sustain constitutive AKT pathway activation. As GCN5 harbours several GSK3β consensus sequences (SXXXS) and can bind with GSK3β, it is believable that the protein level of GCN5 is regulated by the GSK3β kinase activity, but this hypothesis needs more investigation.

The majority of prostate cancer cells are dependent on androgens and activation of AR for growth and survival, and ADT remains the mainstay of treatment for advanced PCa patients.^57^ The PI3K/AKT pathway is often aberrantly activated due to frequent deletion or mutation of the PTEN tumour suppressor gene during prostate tumourigenesis and progression.^34^, ^51^ However, both patient data analysis and preclinical animal model studies invariably show that the loss of PTEN promotes enhanced AKT activity and reduced AR signalling and that inhibition of AKT results in AR activation, while the blockade of AR function increases AKT activities.^58^, ^59^ Here, we showed that K620‐LIFR acetylation could promote the growth and metastasis of PCa cells mainly via activation of AKT signalling. Our findings were verified in both PC3 cells and C4‐2B cells, which were AR negative and AR positive, indicating that the regulatory axis of the LIFR‐K620ac/AKT pathway was independent of AR signalling. Nevertheless, there is still a possibility that the LIFR‐K620ac/AKT pathway could influence AR signalling or be involved in castration resistant prostate cancer, but additional studies are needed in the future to address this.

## 5 CONCLUSION

Conclusively, we first demonstrated that K620 acetylation mediates the formation of LIFR homodimers to further activate the AKT signalling pathway and is upregulated by AKT (thus forming a positive feedback loop); this activity is essential for the malignant progression of PTEN‐deficient PCa. In detail, this study highlights the critical and tightly orchestrated roles of LIFR/PDPK1/AKT/GCN5 in the development of PCa and reveals LIFR‐K620 acetylation as a key hub of the regulation circuit. Mechanistically, we observed that the formation of the LIFR homodimer mediated by K620 acetylation promotes AKT activation by increasing the interaction between AKT and PDPK1 and increases the expression level of the acetyltransferase GCN5 by blocking CRL4^CDT2^ E3 ligase complex‐mediated protein degradation via the ubiquitination‐proteasome pathway, thus further increasing the levels of LIFR‐K620 acetylation and promoting PCa progression in PTEN‐null tumours with a positive feedback loop. By contrast, high levels of GSK3β lead to reduced levels of GCN5 protein and LIFR‐K620 acetylation in PTEN WT tumours (Figure 7I,J).

Our findings uncover high clinical value of the regulatory circuit of LIFR‐K620 acetylation, since most advanced PCa harbours PTEN mutations or overactivation of PI3K/AKT signalling.

## CONFLICT OF INTEREST

The author declares that there is no conflict of interest that could be perceived as prejudicing the impartiality of the research reported.

## Supporting information

Supporting Information
