# Supplementary material for: Leukemia inhibitory factor receptor homodimerization mediated by acetylation of extracellular lysine promotes prostate cancer progression through the PDPK1/AKT/GCN5 axis
Source: Clin Transl Med. 2022 Feb 16;12(2):e676. doi: 10.1002/ctm2.676 (PMC8849371; doi:10.1002/ctm2.676)
Supplement: Supplementary file 1 — Supporting Information [file CTM2-12-e676-s001.docx]

**Supplementary Materials and Methods**

**Antibodies**

Rabbit polyclonal antibody against LIFR (10628-MM07, 1:100 for IHC-P, 1:50 for ELISA) was purchased from Sinobiological (Beijing, China). Rabbit polyclonal or monoclonal antibodies against LIFR (PA5-115511, 1:1000 for WB), GP130 (MA5-34888, 1:1000 for WB), GSK3β (39-9500, 1:1000 for WB), GCN5 (MA5-14884, 1:2000 for WB) and PDPK1 (MA5-31835, 1:2000 for WB) were purchased from Thermo Fisher (Lafayette, CO, USA). Mouse monoclonal anti-actin (MA1-744, 1:5000 for WB), anti-FLAG (f3165, 1: 5000 for WB), anti-HA (h6908, 1: 2500 for WB) and antiKi67 (SAB5300423, 1:500 for IHC) were purchased from Sigma (Lafayette, CO, USA).

Rabbit polyclonal or monoclonal antibodies against AKT (#4691, 1:1000 for WB) and p-AKT-S473 (#[4051](https://www.cellsignal.com/products/primary-antibodies/phospho-akt-ser473-587f11-mouse-mab/4051?site-search-type=Products&N=4294956287&Ntt=akt+473&fromPage=plp) for WB, #[3787](https://www.cellsignal.com/products/primary-antibodies/phospho-akt-ser473-736e11-rabbit-mab/3787?site-search-type=Products&N=4294956287&Ntt=akt+473&fromPage=plp) for IHC and IF), p-AKT-T308 (#4056), YAP (#14074, 1:1000 for WB) and p-YAP-S127 (#4911), GSK3β-S9 (#9336, 1:1000 for WB), S6 (#2217, 1:1000 for WB), and p-S6-S235/236 (#2211, 1:1000 for WB) were purchased from Cell Signaling Technology (Beverly, CA, USA).

LIFR-K378ac, LIFR-K442ac and LIFR-K620ac antibody were generated by Wuhan ABclonal Technology Co. Ltd. Synthesized peptide SYTLVESFSGK(ac)YVRL, PHTPTSFKVK(ac)DINST, NSVGSSPPSK(ac)IASME were coupled with KLH as antigen to immunize rabbit. Serums from immunized rabbits were collected after four doses of injection. High titers of antiserums against LIFR-K378ac, LIFR-K442ac and LIFR-K620ac were obtained in immunized rabbits after affinity purification by acetylated peptides.

**Reagents**

ANTI-FLAG® M2 Affinity Gel (a2220), hematoxylin solution (105175) and eosin (102439)

AKTi, LY294002 (S1105); cycloheximide (CHX, S7418) and MG132 (S2619) were purchased from Sigma (Lafayette, CO, USA) or Selleck (Houston, TX, USA).

GP130/IL6ST siRNA oligos (AM51331) and GCN5 siRNA oligos (AM16708) were purchased from Thermo Fisher (Lafayette, CO, USA). CBP, TIP60, P300, PCAF siRNA oligos were purchased from GenePharma (Shanghai, China).

**Enrichment of soluble LIFR and identification of lysine acetylation by LC-MS/MS**

Briefly, tissue samples and clinical parameters of PCa patients were collected. A 2-ml blood sample was collected from each PCa patient, and the red blood cells or other debris were removed by centrifugation. Protein ultrafiltration tubes with an intercepted molecular weight of 50 kDa were used to reduce the total volume of blood samples and save antibody against LIFR. Blood samples with shortened volumes were diluted with immunoprecipitation (IP) buffer to 50 ml. An 80-μg antibody against LIFR was used to enrich soluble LIFR in a 50-ml IP system. The proteins immunoprecipitated by the LIFR antibody were identified by LC-MS/MS, and acetylation chemical modifications at LIFR-lysine sites were extracted. In detail, the precipitated complexes were boiled at 95°C for 10 min. LIFR was separated from the complexes using SDS-PAGE gel, and the corresponding gel was cut down and processed as described previously (28122243), such as reductive alkylation, trypsin digestion and peptide extraction. The peptides were analyzed by liquid chromatography mass spectrometry (LC-MS/MS) on a Q Exactive mass spectrometer (Thermo Fisher Scientific, Waltham, MA). Proteins were identified by a database search of the fragment spectra against the National SwissProt protein database (EBI) using Mascot Server 2.4 (Matrix Science, London, UK). Acetyl-LIFR peptide matches were analyzed by using MaxQuant v1.5.2.8 implemented in Proteome Discoverer and manually curated.

**DNA constructs and mutagenesis**

PCR-amplified full-length LIFR or the extracellular domain (1-694 aa) was cloned into pCDH, pCDH-3’Flag, pCDH-3’SFB or pCDH-5’HA. PCR-amplified SIRT1, SIRT2, CBP, p300, PCAF, GCN5, TIP60 and PDPK1 were cloned into pCDH-5’HA. LIFR mutations were generated using the QuickChange site-directed mutagenesis kit (Stratagene, La Jolla, CA, USA).

The pLKO.1-shRNA plasmid construct for LIFR was generated with the oligonucleotide sequence 5’-GCCAAAGAATTCTCATGTACT-3’ targeting the 3’UTR region of the LIFR transcripts.

The lentiCRISPRv2-sgRNA plasmid construct for PDPK1 was generated with the oligonucleotide sequence 5’- TCGTCGTCTTCCGACATAGC-3’ targeting the CDS of the PDPK1 transcripts.

**Enzyme-linked immunosorbent assay (ELISA)**

Antibodies against LIFR-K620 acetylation were coated with colloidal gold. We used 0, 10 pM, 200 pM, 4 nM, and 80 nM peptides of LIFR-K620 acetylation, which was used to prepare an antibody against LIFR-K620 acetylation, to generate a standard curve.

The PCa patients were divided into two groups according to metastasis or no metastasis. Fifty microliters of blood serum from each patient was placed in each well with one well for technical repeats. Then, add 50 µl of 1× antiLIFR-K620 acetylation (a total of 500 ng antibody in 50 µl) specific antibody to each well and incubate for 2 hours at room temperature (RT). Then, 50 µl of prepared 1× HRP anti-rabbit IgG (a total of 200 ng antibody in 50 µl) was added and incubated for 1 hour at RT. Then, 100 µl of Ultra TMB ELISA Substrate was added to each well and incubated for 30 min at RT. Then, 50 µl of Stop Solution was added to each well. The optical density of each well was measured at 450 nm in a microplate spectrophotometer, and we read at 450 nm immediately. The OD450 nm value/50 µl is regarded as the patients’ LIFR-K620 acetylation intensity in the blood samples.

**Cell culture and transfection**

Cells were all purchased from American Type Culture Collection or Cell Bank, Shanghai Institutes for Biological Sciences, Chinese Academy of Sciences. PC3 and DU145 cells were cultured in standard RPMI or DMEM, respectively, supplemented with 10% fetal bovine serum. Cell lines were tested for c-Mycoplasma contamination before use to ensure that they were mycoplasma-free. Plasmids were transfected into PCa cells using FuGENE transfection reagent (Life Technologies, USA), while a nonspecific plasmid (shNT or Vec) was used as an experimental control (CTRL).

Cells were transfected with siRNA duplexes (60-100 nM) by using Lipofectamine (Invitrogen) or Dharmacon Transfection reagents (Dharmacon) according to the manufacturer’s instructions. To establish individual stable cells, retrovirus/lentivirus was used. Standard 24-well Boyden invasion chambers (BD Biosciences) were used to assess cell migration abilities. For the soft agar colony formation assay, cells were suspended in RPMI 1640 containing 0.35% low-melting agar (Invitrogen) and 10% FBS and seeded onto a coating of 0.7% low-melting agar in RPMI 1640 containing 10% FBS. The results were calculated based on 3 independent experiments. For inhibitor treatments, please see descriptions in the figure legends.

**Lentivirus package and infection**

Amplification of lentivirus was via standard methods in subconfluent HEK293T cells. Infection of prostate cancer cell lines was performed in the presence of polybrene (Sigma) at a final concentration of 8 µg/ml. Cells were incubated with lentivirus mixture for 72 hours, digested with trypsin in fresh growth medium, and then sorted with green fluorescence for stable expression or knockdown. The constructed stable cell lines were amplified and saved for future experiments.

**PDPK1 Knockout Cell Line Construct**

LentiCRISPRv2-sgRNA plasmid of PDPK1 was packaged into lentivirus and infected with PC3 cells. After 72 hours of infection, the cells were added 2 μg/mL puromycin for screening the monoclonal cells. The positively screened cells were digested, and then 400 single cells were counted and seeded in a 10 cm culture dish. Single cell colonies were obtained after about 1 week, and the cell pellet was picked up under a microscope and inoculated into a 24-well plate to continue the culture. Total proteins were extracted and the expression of PDPK1 was detected by Western blot.

**Coimmunoprecipitation and Western blot (WB)**

Immunoprecipitation was performed with lysates from the indicated cultured cells, followed by immunoblotting with the corresponding antibodies. Briefly, after trypsinizing, the cells were harvested and washed twice with cold PBS. Cell pellets were resuspended and lysed in buffer (50 mM Tris-HCl, pH 7.4, 150 mM NaCl, 1% Triton X-100, 5 mM EDTA, 1 mM NaVO_3_, 50 mM NaF and protease inhibitor cocktail). Then, the lysates were centrifuged at 13,000 g to remove the cell debris. The supernatant was transferred to a prechilled microcentrifuge tube. Protein concentration was determined using the BCA Protein Assay Kit (Pierce) according to the manufacturer’s instructions. One milligram of protein was incubated with the indicated antibodies overnight and then mixed with protein A or protein G-agarose beads (Santa Cruz, USA).

Immunocomplexes were collected by centrifugation at 1000 g, resolved on SDS-PAGE gels and subsequently transferred to polyvinylidene fluoride membranes (Millipore). The blots were blocked with 5% nonfat milk followed by incubation with primary antibodies and HRP-conjugated secondary antibodies. Blots were developed using SuperSignal West Pico (Thermo Scientific, USA) and detected by a Tanon 6600 Luminescent Imaging Workstation.

**Proteomic profiling and quantitative RT-PCR (qRT-PCR)**

The proteins of 10^6^ cells were extracted and identified by LC-MS/MS. In detail, the cells were lysed by RIPA buffer and boiled at 95°C. Lysates were separated using SDS-PAGE gel and the whole lane was cut down and processed as described previously[1], such as reductive alkylation, trypsin digestion and peptide extraction. The peptides were analyzed by liquid chromatography mass spectrometry (LC-MS/MS) on a Q Exactive mass spectrometer (Thermo Fisher Scientific, Waltham, MA). Proteins were identified by a database search of the fragment spectra against the National SwissProt protein database (EBI) using Mascot Server 2.4 (Matrix Science, London, UK). Total RNA was extracted with an RNA high-purity total RNA rapid extraction kit (QIAGEN, 74034), and RNA profiling and data collection and analysis were performed in Decode Gene (Shanghai, China). cDNA was prepared using a GoScript reverse transcription system (Promega, TM316). qRT-PCR was performed using SYBR PCR premixture (Promega, M7502) under the following conditions: 5 min at 95°C followed by 38 cycles at 95°C for 30 s, 60°C for 40 s, and 72°C for 1 min using an ABI 7500 fast system. Data were normalized to the expression of the control gene (β-actin) for each experiment. Data represent the mean ± SD of three independent experiments. The sequences of primer pairs used for qRT-PCR are listed in Supplementary Table 5.

1. Wang, X.J., et al., *Opposing Roles of Acetylation and Phosphorylation in LIFR-Dependent Self-Renewal Growth Signaling in Mouse Embryonic Stem Cells.* Cell Rep, 2017. **18**(4): p. 933-946.

**Supplemental Table 1**

Information of PCa patients and control group for blood test

| Variables | Control Group | PCa Patients Group |
| --- | --- | --- |
| Numbers | 29 | 46 |
| Age,yr. | 35-71 | 39-83 |
| Preoperative PSA,ng/ml | 0.50-15.00 | 1.20-32.00 |
| Family PCa history | 4(13.79) | 7(15.22) |
| Pathologic events | N (%) | N (%) |
| BPH | 21(72.41) |  |
| Prostatitis | 6 (20.69) |  |
| None | 2 (7.90) |  |
| Primary, no invasion |  | 28(60.87) |
| Seminal vesicle invasion |  | 13(28.26) |
| Lymph node invasion |  | 5(10.87) |
| Positive surgical magins |  | 11(23.91) |

**Supplemental Table 2**

Information of Asian radical prostatectomy cohort

| Variables | All patients |
| --- | --- |
| Numbers | 261 |
| Age at diagnosis,yr. | 55-85 |
| Year of surgery | 2008-2010 |
| Preoperative PSA,ng/ml | 9.2 (1.00-28.00) |
| No.of bichemical recurrence | 126 (48.27) |
| Pathologic gleason score, | N (%) |
| <6 | 61 (23.37) |
| 7 | 132 (50.57) |
| 8 | 38 (14.56) |
| >9 | 30 (11.49) |
| Adverse pathologic events, | N (%) |
| Seminal vesicle invasion | 28 (10.72) |
| Lymph node invasion | 6 (2.30) |
| Positive surgical magins | 24 (9.19) |

**Supplemental Table 3**

Univariate and multivariate cox analysis of LIFR-K620ac, PTEN, PSA and other parameters in BCR

| **Variables** | **Univariate Cox** | | **Multivariate Cox** | | |
| --- | --- | --- | --- | --- | --- |
|  | **HR (95% CI)** | ***P* value** |  | **HR (95% CI)** | ***P* value** |
| Gleason Score | 2.13 (1.51 to 3.26 ) | 0.032 |  | 1.68 (1.12 to 2.56) | 0.011 |
| PSA | 2.41 (1.22 to 2.96 ) | 0.006 |  | 1.98 (1.08 to 3.55) | 0.048 |
| LIFR-K620ac | 2.79 (1.73 to 3.22) | 0.001 |  | 2.65 (1.43 to 3.86) | 0.003 |
| PTEN | 0.53 (0.38 to 0.87) | 0.019 |  | 0.67 (0.45 to 1.03) | 0.054 |
| P-AKT | 2.84 (1.27 to 3.72) | 0.008 |  | 2.23 (1.57 to 4.12) | 0.001 |
| Age | 1.08 (1.02 to 1.31) | 0.003 |  | 1.03 (0.95 to 1.22) | 0.082 |

**Supplemental Table 4**

Univariate analysis of LIFR-K620ac, PTEN and PSA in different clinicopathological group

| **Univariate Cox** | **Clinicopathological Group** | **Log-rank p value** | **HR** | **95% Cl** |
| --- | --- | --- | --- | --- |
| LIFR-K620ac | Gleason Score (≤6) | 0.022 | 2.18 | 1.17 to 3.15 |
|  | Gleason Score (7-8) | 0.007 | 2.26 | 1.26 to 3.47 |
|  | Gleason Score (≥9) | <0.001 | 2.58 | 1.82 to 3.98 |
| PTEN | Gleason Score (≤6) | 0.003 | 0.35 | 0.22 to 0.62 |
|  | Gleason Score (7-8) | 0.015 | 0.47 | 0.38 to 0.79 |
|  | Gleason Score (≥9) | 0.048 | 0.79 | 0.51 to 0.95 |
| PSA | Gleason Score (≤6) | 0.028 | 1.69 | 1.12 to 2.96 |
|  | Gleason Score (7-8) | 0.012 | 2.12 | 1.49 to 3.27 |
|  | Gleason Score (≥9) | <0.001 | 2.49 | 1.51 to 3.73 |

**Supplemental Table 5**

| Primer Name: | Sequence |
| --- | --- |
| siRNA oligos for CBP: | 5’-AUCGCCACGUCCCUUAGUAAC-3’ |
| siRNA oligos for P300: | 5’-AUACUCAGCCGGAGGAUAUUU-3’ |
| siRNA oligos for PCAF: | 5’-GUUGGCUAUAUCAAGGAUUAU-3’ |
| siRNA oligos for Tip60: | 5’-CAAGUGUCUUCAGCGUCAUUU-3’ |
| siRNA oligos for GCN5: | 5’-GCGCAUGCCUAAGGAGUAUAU-3’ |
| qPCR Forward Primer for CBP: | 5’-CGGCTCTAGTATCAACCCAGG-3’ |
| qPCR Reverse Primer for CBP: | 5’- TTTTGTGCTTGCGGATTCAGT-3’ |
| qPCR Forward Primer for p300: | 5’- GCTTCAGACAAGTCTTGGCAT-3’ |
| qPCR Reverse Primer for p300: | 5’- ACTACCAGATCGCAGCAATTC-3’ |
| qPCR Forward Primer for PCAF: | 5’- TTTGTCTACCTCCATCTCAACCT-3’ |
| qPCR Reverse Primer for PCAF: | 5’- GACGGACCACGTACTTGTTATC-3’ |
| qPCR Forward Primer for Tip60: | 5’- GACGGAAGCGAAAATCGAATTG-3’ |
| qPCR Reverse Primer for Tip60: | 5’- GGTGCTGACGGTATTCCATCA-3’ |
| qPCR Forward Primer for GCN5: | 5’- GCAAGGCCAATGAAACCTGTA-3’ |
| qPCR Reverse Primer for GCN5: | 5’- TCCAAGTGGGATACGTGGTCA-3’ |
| shRNA for LIFR Forward Primer : | 5’CCGGGCCAAAGAATTCTCATGTACTCTCGAGAGT-ACATGAGAATTCTTTGGCTTTTTG-3’ |
| shRNA for LIFR Reverse Primer : | 5’AATTCAAAAAGCCAAAGAATTCTCATGTACTCTC-GAGAGTACATGAGAATTCTTTGGC-3’ |
| sgRNA for PDPK1: | 5’- TCGTCGTCTTCCGACATAGC-3’ |

**Supplementary Figures and Legends**


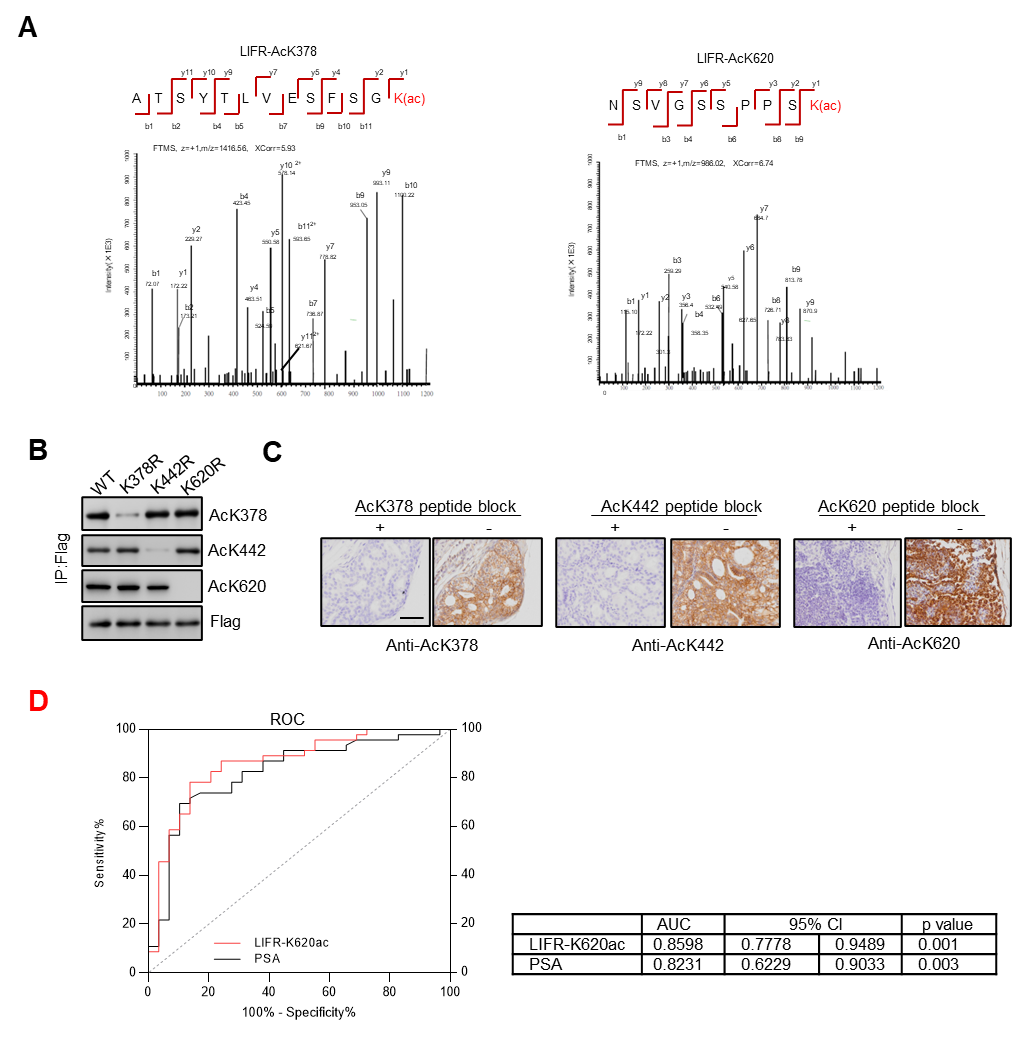


**Supplementary Figure 1.** (**A**) Mass spectrometry analysis of a tryptic fragment at m/z 1416.56 (mass error was 2.28 ppm) matched to the doubly charged peptide 366-ATSYTLVESFSG_ac_K-378, suggesting that K378 was acetylated. The Sequest score for this match was Xcorr = 5.93; Mascot scores were 61, expectation value 7.6×10^-4^. The presence of the y_1_ at 172.22 indicates that the K378 residue is acetylation modified (Left, A). Mass spectrometry analysis of a tryptic fragment at m/z 986.02 (mass error was 1.76 ppm) matched to the doubly charged peptide 611-NSVGSSPPS_ac_K-620, suggesting that K620 was acetylated. The Sequest score for this match was Xcorr = 6.74; Mascot scores were 55, expectation value 6.1×10^-4^. The presence of the y_1_ at 172.22 indicates that the K620 residue is acetylation modified (Right, A). The probability of Ack378 was 58.24% while the probability of AcK620 was 64.61%. (**B-C**) the specificity of these antibodies against LIFR-AcK378, LIFR-AcK442 or LIFR-AcK620 were ascertained by western blot or IHC. Scale bars:100 μm. (D) ROC curve and analysis of LIFR-K620ac and PSA in PCa diagnosis. Statistics was performed by Shapiro-Wilk test.


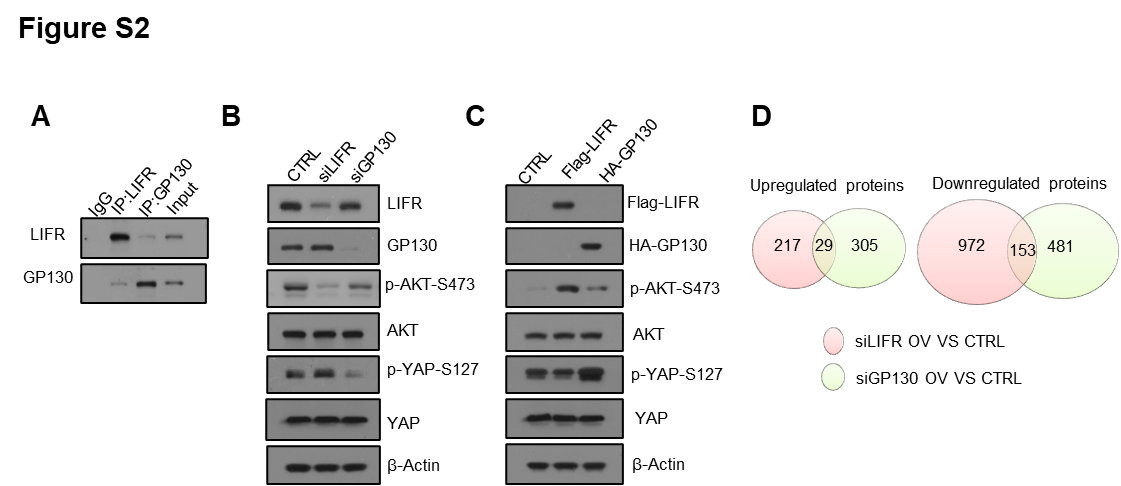


**Supplementary Figure 2.** (**A**) Using antibodies against LIFR or GP130, a Co-IP assay between LIFR and GP130 in PC3 cells was performed. (**B**) Transiently depleting LIFR or GP130 in PC3 cells, AKT and YAP activity were tested by western blot using indicated antibodies. (**C**) Transiently overexpressing LIFR or GP130 in PC3 cells, AKT and YAP activity were tested by western blot using indicated antibodies. (**D**) The comparative proteomic profiling without labeling to quantify the differential proteins using siLIFR, siGP130 and CTRL cells. overall number of significantly changed proteins regulated by LIFR or GP130 were shown.


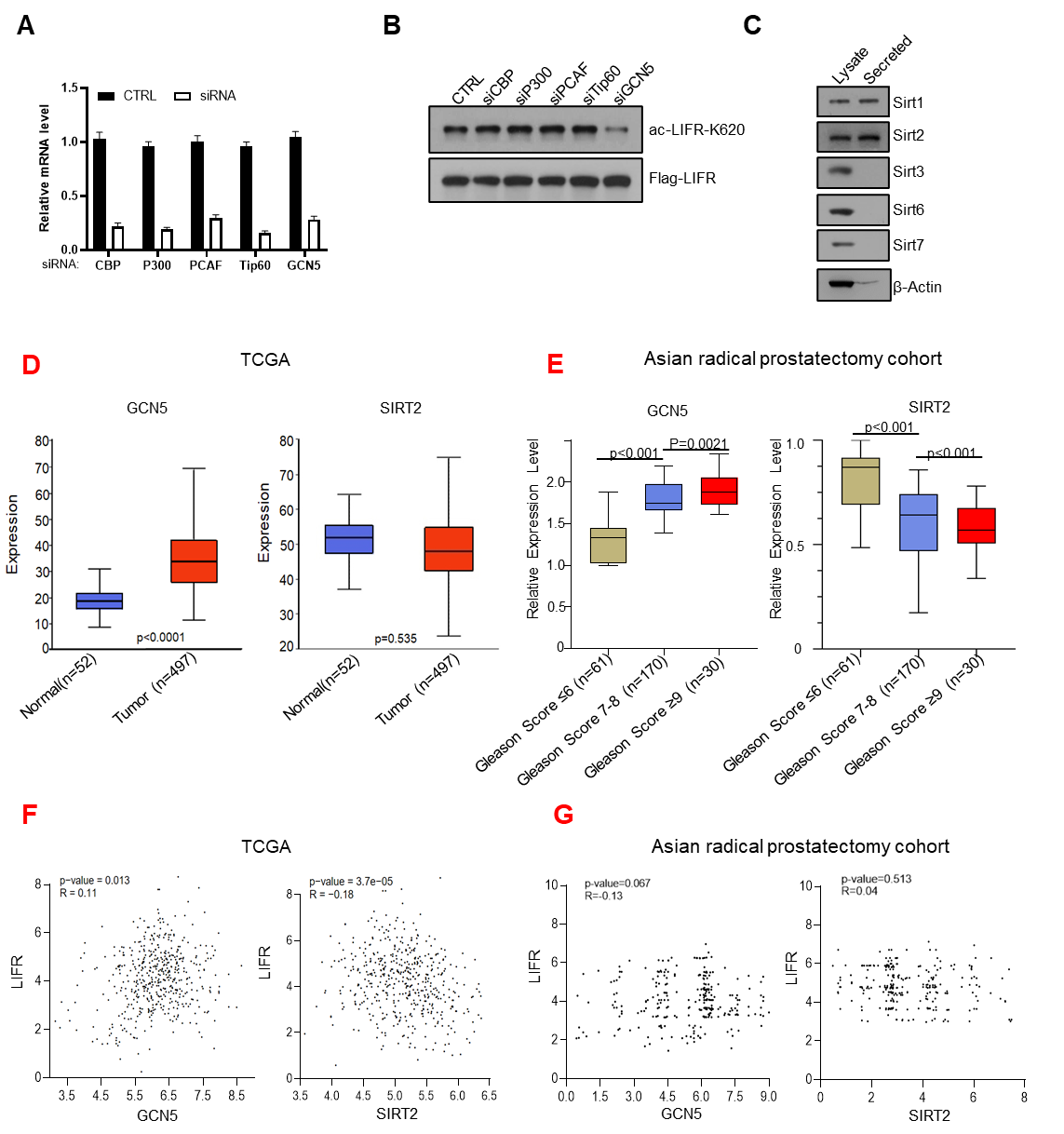
**Supplementary Figure 3.** (**A**) The relative mRNA level of indicated genes by respective siRNA oligos treatment in PC3 cells. (**B**) Western blot analysis of lysates from PC3 cells treated with siRNA oligos of indicated genes using antibodies against LIFR-K620ac and Flag-LIFR. (**C**) Lysates from whole cells or supernatant were analyzed by western blot against indicated antibodies. (**D-E**) GCN5 and SIRT2 expression in TCGA database or Asian radical prostatectomy cohort. p values were calculated by two-tailed Student’s t test. (F-G) Pearson’s correlation coefficient was used to test for correlations of GCN5 or SIRT2 with LIFR in TCGA database or the IHC results of Asian radical prostatectomy cohort.

**
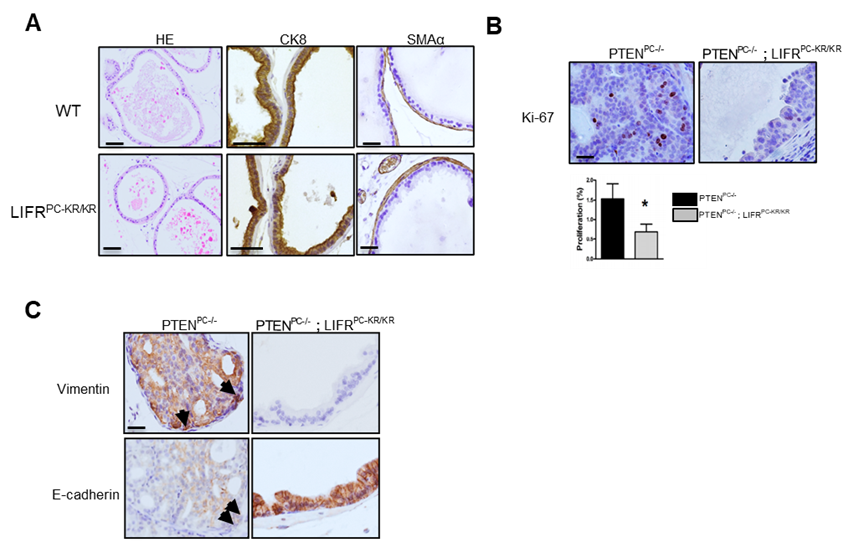
**

**Supplementary Figure 4.** **LIFR-K620 acetylation is required for PCa progression.** (**A**) Immunohistochemical analysis of CK8 and p63 positive cells in prostate epithelium of wild-type and LIFR^PC-KR/KR^ mice at the age of 8 months. (**B**) Ki-67 staining of prostate sections from 4-month-old PTEN^PC–/–^ and PTEN^PC–/–^ LIFR^PC-KR/KR^ mice, and semi-quantitative results are shown in the right panel. Results are presented as mean ± SEM. (**C**) Immunohistochemical analysis of E-cadherin and Vimentin positive cells in prostate epithelium of 8-month-old PTEN^PC–/–^ and PTEN^PC–/–^ LIFR^PC-KR/KR^ mic *: p < 0.05. Scale bars: 100μm (A), 50μm (B and C).


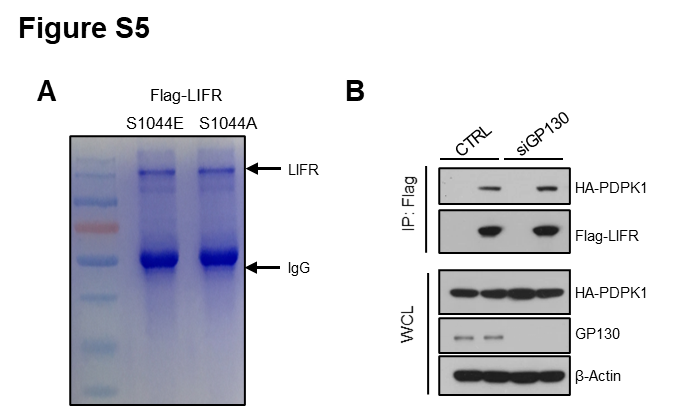


**Supplementary Figure 5.** (**A**) Purification of FLAG-LIFR (S1044E and S1044A) was examined by SDS-PAGE and stained by Coomassie Brilliant Blue. (**B**) FLAG-LIFR and HA-PDPK1 were co-transfected into PC3 cells with or without GP130. M2 FLAG beads were used to enrich FLAG-LIFR associated proteins which were examined by indicated antibodies.


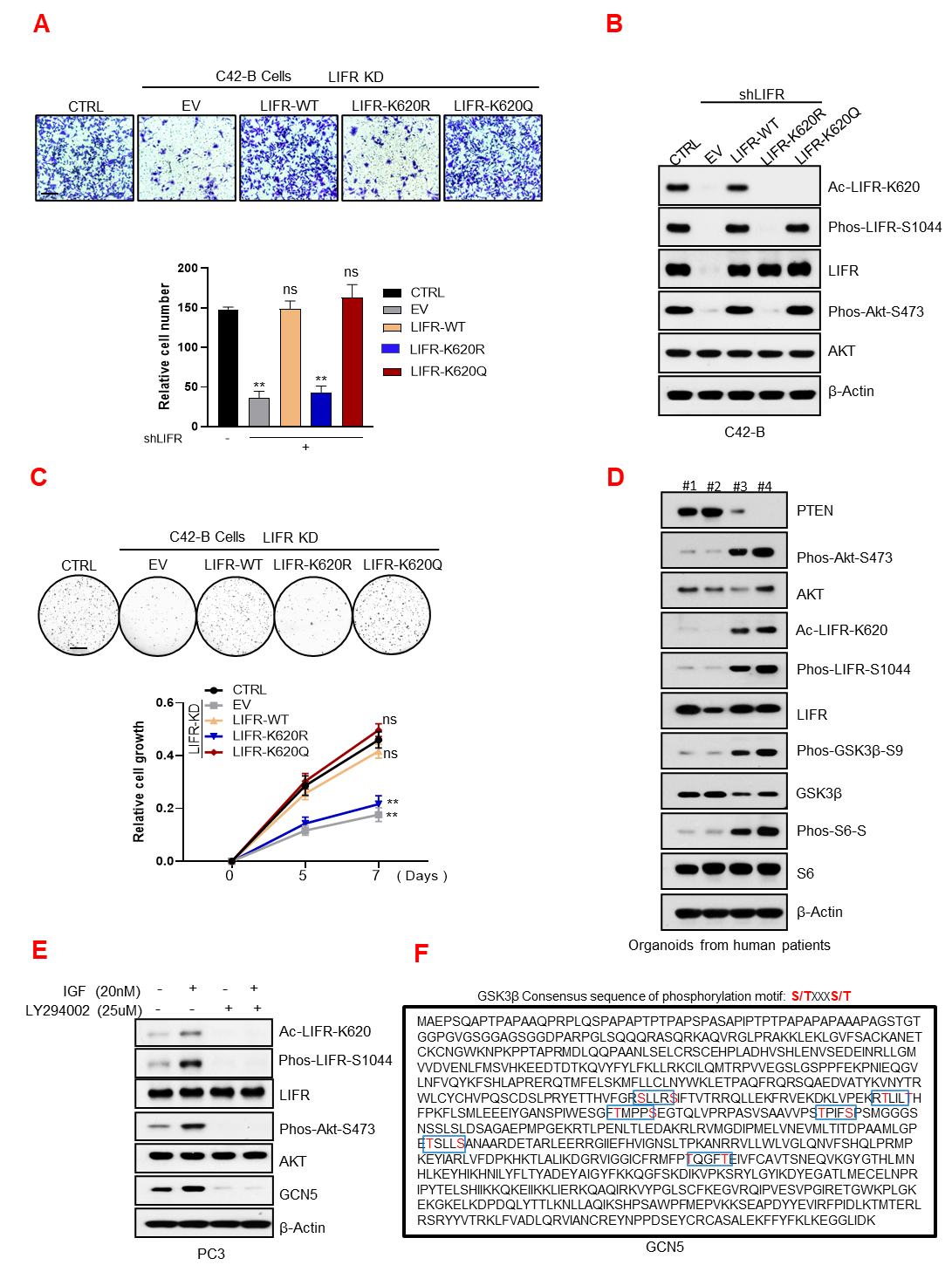


**Supplementary Figure 6.** (**A**). Transwell images(upper) and quantification (bottom) of C42-B cells with LIFR knockdown and LIFR mutants rescued. (**B**). WB analysis of C42-B cells with LIFR knockdown and LIFR mutants rescued using indicated antibodies. (**C**). Colony formation(upper) and growth curve(bottom) of C42-B cells with LIFR knockdown and LIFR mutants rescued. (**D**). WB analysis of the organoid samples from the 4 patients using indicated antibodies. (**E**). WB analysis of IGF (20 nM) or LY294002 (25 uM) treated PC3 cells for 24 hours by indicated antibodies. (**F**). The GSK3β Consensus sequence of phosphorylation motif analysis of GCN5. p values were calculated by two-tailed Student’s t test. **p < 0.01. ns, not statistically significant. Scale bar: 100 μm (B), 800 μm (D).
